# A systems biology approach reveals a link between systemic cytokines and skeletal muscle energy metabolism in a rodent smoking model and human COPD

**DOI:** 10.1186/s13073-014-0059-5

**Published:** 2014-08-09

**Authors:** Peter K Davidsen, John M Herbert, Philipp Antczak, Kim Clarke, Elisabet Ferrer, Victor I Peinado, Constancio Gonzalez, Josep Roca, Stuart Egginton, Joan A Barberá, Francesco Falciani

**Affiliations:** Centre for Computational Biology and Modelling, Institute for Integrative Biology, University of Liverpool, Crown Street, L69 7ZB Liverpool, UK; School of Immunity and Infection, University of Birmingham, Birmingham, UK; Department of Pulmonary Medicine, Hospital Clinic, University of Barcelona, Barcelona, Spain; Institut d’Investigacions Biomédiques August Pi i Sunyer (IDIBAPS), Barcelona, Spain; Biomedical Research Networking Center on Respiratory Diseases (CIBERES), Barcelona, Spain; Department of Biochemistry and Molecular Biology and Physiology, University of Valladolid, Valladolid, Spain; School of Biomedical Sciences, Faculty of Biological Sciences, University of Leeds, Leeds, UK

## Abstract

**Background:**

A relatively large percentage of patients with chronic obstructive pulmonary disease (COPD) develop systemic co-morbidities that affect prognosis, among which muscle wasting is particularly debilitating. Despite significant research effort, the pathophysiology of this important extrapulmonary manifestation is still unclear. A key question that remains unanswered is to what extent systemic inflammatory mediators might play a role in this pathology.

Cigarette smoke (CS) is the main risk factor for developing COPD and therefore animal models chronically exposed to CS have been proposed for mechanistic studies and biomarker discovery. Although mice have been successfully used as a pre-clinical *in vivo* model to study the pulmonary effects of acute and chronic CS exposure, data suggest that they may be inadequate models for studying the effects of CS on peripheral muscle function. In contrast, recent findings indicate that the guinea pig model (*Cavia porcellus*) may better mimic muscle wasting.

**Methods:**

We have used a systems biology approach to compare the transcriptional profile of hindlimb skeletal muscles from a Guinea pig rodent model exposed to CS and/or chronic hypoxia to COPD patients with muscle wasting.

**Results:**

We show that guinea pigs exposed to long-term CS accurately reflect most of the transcriptional changes observed in dysfunctional limb muscle of severe COPD patients when compared to matched controls. Using network inference, we could then show that the expression profile in whole lung of genes encoding for soluble inflammatory mediators is informative of the molecular state of skeletal muscles in the guinea pig smoking model. Finally, we show that CXCL10 and CXCL9, two of the candidate systemic cytokines identified using this pre-clinical model, are indeed detected at significantly higher levels in serum of COPD patients, and that their serum protein level is inversely correlated with the expression of aerobic energy metabolism genes in skeletal muscle.

**Conclusions:**

We conclude that CXCL10 and CXCL9 are promising candidate inflammatory signals linked to the regulation of central metabolism genes in skeletal muscles. On a methodological level, our work also shows that a system level analysis of animal models of diseases can be very effective to generate clinically relevant hypothesis.

**Electronic supplementary material:**

The online version of this article (doi:10.1186/s13073-014-0059-5) contains supplementary material, which is available to authorized users.

## Background

Chronic obstructive pulmonary disease (COPD), one of the top five deadliest diseases worldwide [1], is an inflammatory condition of the lungs that predominantly affects people with a long history of cigarette smoking (CS) [2]. In addition to the clinical manifestations in the lung, COPD is also associated with several extra pulmonary manifestations. Skeletal muscle wasting and dysfunction is one of the most severe of these pathologies [3]. This muscular deconditioning, which is partly independent of the severity of airflow limitation, is a prominent contributor to exercise intolerance [4] as well as being an independent predictor of morbidity and mortality [5]. Long-term CS exposure has a clear potential to contribute to the systemic effects of COPD, as similar findings have been observed in healthy smokers (for example, a decrease in lean muscle mass and force reduction) [6]. However, the direct effects of CS on peripheral muscle function, at the molecular level, are poorly understood.

Although we do not still fully understand the mechanisms that contribute to muscle dysfunction in COPD, there is evidence that multiple factors are likely to influence clinical outcome, such as systemic inflammation, reduced capillary density, tissue hypoxia and subsequent oxidative stress [7]. However, it is not known to what extent increased levels of inflammatory cytokines play a role in muscle wasting. Likewise, hypoxaemia has been linked to several drivers of muscle dysfunction in COPD such as downregulation of energy-consuming processes (for example, protein synthesis, mitochondrial respiration), impaired myogenesis, fibre type shifting [8] and increased serum levels of cytokines [9], but the exact molecular mechanisms through which chronic or intermitted hypoxia affects muscle maintenance are unclear.

Animal models, particularly mouse models, are widely used to study the effects of acute and chronic CS. Importantly, long-term CS exposure in rodent models may be the best approximation of the more acute aspects of lung responses in human COPD [10]. However, the current literature suggests that mice chronically exposed to CS develop either none or only mild peripheral muscle dysfunction [6,11,12]. For example, only two studies have to our knowledge reported a significant decrease in hindlimb muscle weight following long-term whole-body exposure using very high smoking doses (≥20 cigarettes/day) [11,13].

The guinea pig (*Cavia porcellus*), one of the most popular animal models to study infectious diseases [14], has been shown to tolerate CS exposure without the rapid weight loss observed in other pre-clinical models [15]. However, promisingly long-term CS-exposed guinea pigs fail to appropriately gain body weight compared to age-matched sham controls [6,16]. Further, in accordance with previous findings in COPD patients [6], guinea pigs demonstrate CS-induced oxidative stress in limb muscles within 3 months of exposure [6,16], potentially highlighting the relevance of the guinea pig model for studying extrapulmonary co-morbidities that characterise human COPD.

However, the guinea pig is a rather challenging model organism to determine the molecular response due to the lack of a fully annotated genome. This paucity of genetic information is unfortunate since gene expression profiling has shown to be a very promising approach to formulate hypotheses on complex molecular mechanisms underlying pathology [17,18]. Here, we report the development of the first transcriptome-sequencing for guinea pigs representing lung and skeletal muscles, as well as the development and validation of a novel genome-wide microarray platform. With this novel platform, we profiled the transcriptional response of lung and muscle tissues chronically exposed to CS, hypoxia (CH) or to combined stimuli (CSCH). The overarching aim of our study was to assess whether guinea pig hindlimb muscles (both oxidative and glycolytic) show a transcriptional response to these exposures, and whether such gene signatures can be correlated to the expression of lung secreted proteins.

We discovered that indeed skeletal muscles of guinea pigs exposed to all experimental interventions accurately mimic the transcriptional state of human limb muscle sampled from COPD patients with muscle atrophy. Using a relatively simple network inference method we then identified systemic cytokines whose mRNA and serum protein levels are inversely correlated with the transcriptional state of energy metabolism pathways in guinea pig and human COPD patients, respectively.

These results provide further evidence for the utility of the guinea pig smoking model to study muscle wasting in COPD and support the hypothesis that systemic inflammation plays an important role in altering the energy metabolic state in peripheral muscles causing them to dysfunction.

## Methods

### Guinea pig smoking model

Sixteen male Hartley guinea pigs were divided into four groups: one group was exposed to CS for 3 months (n = 4); a second group was kept in normoxia for 10 weeks and subsequently placed in an hypoxic environment (12% O_2_) for 2 weeks (n = 4); a third group (n = 4) was CS-exposed for 3 months and to chronic hypoxia the last 2 smoking weeks; finally we included a fourth group (n = 4) of sham controls remaining in normoxia for the whole study period. To avoid problems related to ageing, young adults were used (8 weeks of age), and to avoid scaling effects body mass was similar (approximately 300 g/animal). All procedures involving animals and their care were approved by the Ethics Committee of the University of Barcelona and by the University of Valladolid Institutional Committee for Animal Care and Use, and were conducted following institutional guidelines that comply with national (Generalitat de Catalunya decree 214/1997, DOGC 2450) and international (Guide for the Care and Use of Laboratory Animals, National Institutes of Health, 85-23, 1985) laws and policies.

Whole lung as well as soleus and lateral gastrocnemius hindlimb muscles were isolated from each animal at the end of the study period. The soleus muscle and the lateral gastro were selected to represent oxidative and glycolytic muscles, respectively. It should be noted that although the gastrocnemius as a whole is a mixed muscle, the lateral portion is predominantly glycolytic.

Animals receiving CS were daily exposed to the smoke of four cigarettes (2R4F; Kentucky University Research; Lexington, KY, USA, 11 mg tar, 0.8 mg nicotine per cigarette), 5 days/week using a nose-only inhalation system (Protowerx Design Inc; Langley, BC, Canada). Sham-exposure to CS was done daily by placing control animals in the nose-only exposure chamber for the same duration (1 h) without cigarettes being lighted. In this experimental model, neither nutritional status (determined via measurements of plasma cholesterol, protein and lipids) nor whole-body weight gain at the end of the study period is significantly different between CS-exposed and sham animals [16]. Moreover, no changes in the proportion of Type I and Type II fibres can be detected between CS-exposed and shams [6]. Detailed information on exposure protocols, pulmonary function data and histological assessments from this study, which demonstrate that observations in lung function and pulmonary structural changes of COPD patients are indeed replicated in the CS-exposed guinea pigs, have been reported in two separate publications [15,19].

### RNA isolation from guinea pig samples

Total RNA was extracted using the RNeasy Mini extraction kits (Qiagen, USA) according to the manufacturer’s recommendations. RNA purity and quality was evaluated using a NanoDrop (Thermo Scientific) and a BioAnalyzer 2100 instrument (Agilent Technologies), respectively. All samples had a RIN score >7.

### Definition of the guinea pig transcriptome by mRNA sequencing and microarray design

In 2008 the guinea pig genome was sequenced to a depth of approximately 7X full coverage, and last updated in 2010. However, because of the lack of cDNA and protein resources the guinea pig genome is at present poorly annotated. Thus, in order to address this issue we performed an in-depth mRNA sequencing of the lung and skeletal muscles transcriptomes and used this to annotate the available guinea pig genome for transcribed sequences. We then used this information to design and validate the custom Agilent microarray platform used in this study.

Transcriptome sequencing was performed using Illumina sequencing. Briefly, NCBI and Ensembl transcripts of guinea pig were combined with transcripts constructed from Illumina paired end reads using the TopHat and Cufflinks algorithms [20,21]. Microarray probe sequences were then chosen based on the combined transcriptome assembly. The raw RNAseq data have been deposited at the Gene Expression Omnibus (GEO) under the reference number GSE56099. A full description of the procedure is provided in Additional file 1.

### Guinea pig microarray gene expression profiling

One hundred nanograms of total RNA from each sample was amplified and converted into labelled cRNA using Agilent’s Low Input Quick Amp Labelling Kit according to the manufacturer’s recommendations. Cy3-labelled cRNA (600 ng/sample) was hybridised to our custom *Cavia porcellus* oligonucleotide microarray (manufactured by Agilent) in randomised sample order, which generated 61,657 measures per sample (18,073 annotated genes). Hybridisation, washing and scanning of arrays were performed according to manufacturer’s protocol. Three samples (one from each tissue) were lost during the process of generating raw data. All scanned microarrays passed all 11 of the Agilent’s quality metrics. Capture probes that were flagged (that is, did not pass Agilent’s ‘well above background’ condition) on at least 80% of the chips were removed prior to data analysis, such that only those capture probes with a raw signal greater than 99% of the background population signal, for at least 20% of the samples, were retained (29,333 probes were discarded).

Raw microarray data were then normalised against sham controls for each of the three tissues using loess in the ‘marray’ [22] and ‘limma’ [23] Bioconductor packages. Arrays were examined using hierarchical clustering and principal component analysis (PCA) to identify outliers prior to statistical analysis.

The statistical significance of differential expression of each gene was determined using the significance analysis of microarray (SAM) algorithm [24] with a false discovery rate (FDR) cutoff of 1%. Gene ontology (GO) and Kyoto Encyclopedia of Genes and Genomes (KEGG) enrichment in differentially expressed genes was examined using the web-based tool DAVID [25]. Disease KEGG pathways were excluded from the analysis to maximise biological interpretability. Therefore the analysis was restricted to KEGG group 1-4 (Metabolism, Genetic Information Processing, Environmental Information Processing and Cellular Processes). The microarray data have been deposited in GEO under accession number GSE56099.

### RT-qPCR validation of custom guinea pig array

Reverse transcription of 1 ug of isolated total RNA from whole-lung tissue (same RNA as were used for the microarray part) was performed using the Tetro cDNA synthesis kit (Bioline) with random hexamer primers following the manufacturer’s instructions. The resulting cDNA was diluted 10-fold and 2.5 uL of this was used to perform qPCR in triplicate (25 uL reaction mixture volume) using the Maxima SYBR green (Thermo Scientific) and 300 nM of primers according the manufacturer’s instructions. To adjust for variations in the cDNA synthesis, each gene was normalised to that of 18S ribosomal RNA and beta-actin mRNA, respectively. All reactions were run in singleplex on a StepOnePlus Real Time System (Applied Biosystems) at 95°C for 10 min, followed by 40 cycles at 95°C for 15 s and 60°C for 1 min. Two-fold dilution series were performed for all primer pairs to verify the linearity of the assay. In addition, dissociation curve analysis was performed after each PCR to check for unspecific signals. Quantification was performed using the comparative cycle threshold (2^-∆∆Ct^) method.

The following primers were used: CXCL9 fwr: 5′-AGGCACCCCAGTAATGAG-3′; CXCL9 rev: 5′-TGATTTCTGTTTTCTCACACG-3′; CXCL10 fwr: 5′-TCTGAGTGGGACTCAAGGAATACC-3′; CXCL10 rev: 5′-TCCAGACATCTCTTCTCCCCATTC-3′; beta-actin fwr: 5′-GAGGCACCAGGGAGTCATG-3′; beta-actin rev: 5′-AAGGTGTGGTGCCAGATCTTCTC-3′; 18S rRNA fwr: 5′-GTACAGTGAAACTGCGAATGGCTC-3′; 18S rRNA rev: 5′-CCGTCGGCATGTATTAGCTCTAG-3′.

### Human COPD clinical studies

In order to assess the clinical relevance of the findings in respect to the guinea pig dataset, we took advantage of a human microarray dataset we have previously published [26]. This defined the baseline/resting transcriptional state of the *vastus lateralis* muscle in severe COPD patients with either a normal (n = 9) or low (n = 6) body mass index (BMI) and healthy controls matched for age and smoking history (n = 12). In addition, the low BMI COPD group also had a significantly lower fat free mass index (FFMI) (on average 16.7 kg/m^2^; Additional file 2), a clear surrogate for muscle wasting. All participants signed a written, informed consent approved by the Ethics Committee on Investigations Involving Human Subjects at the Hospital Clinic, Universitat de Barcelona, and the study was conducted in accordance with principles of the Declaration of Helsinki. Briefly, raw Affymetrix CEL files were RMA normalized following removal of probes that were termed ‘absent’ in more than 80% of the samples by the MAS5 algorithm inside the affy package (26,197 probes were discarded). Following probe summarization, a two-class unpaired SAM analysis was performed using the R package ‘samr’ comparing gene expression levels between COPD patients with a muscle wasting phenotype and matched controls. Enrichment of KEGG terms (group 1 to 4) in the resulting gene lists was assessed using DAVID. Enriched terms used to define the ‘true response’ in the cross-species overlap analysis were defined as having an EASE score *P* value <0.2. Further, detailed demographic data on the human subjects, including pulmonary measurements are provided in Additional file 2. The raw microarray CEL files are deposited under the reference number GSE27536.

In addition, we also analysed a public microarray dataset published by the Ronald Crystal lab [27] examining transcriptional changes in small airway epithelium from healthy non-smokers (n = 47), healthy smokers (n = 58) and smokers with COPD (n = 22), respectively (GSE19407). Due to a clear scan date batch issue (Additional file 3, Figure A), we focused our analysis on the data generated in years 2006 and 2007 (hence excluding the two samples scanned in 2005 as well as the 36 samples processed in 2008). As both human studies were conducted on the Affymetrix U133 + 2 platform, the data analysis strategy of the raw CEL files representing the pulmonary data was identical to that of the human dataset in skeletal muscle presented in this paper (see above) (see Additional file 4 for the list of regulated transcripts with fold-changes as well as functional enrichment analysis).

### Summarising the molecular state of skeletal muscle using indices of pathway transcriptional activity

In order to reduce the complexity of the genome-wide transcriptional state of guinea pig skeletal muscles, thereby increasing statistical power, we computed indices of the overall pathway transcriptional activity [28,29]. For each of the two guinea pig hindlimb muscles, we first mapped the thousands of individual gene expression measures onto KEGG pathways using DAVID [30]. We then summarised the transcriptional activity for the enriched pathways (FDR <10%) by computing the first three principal components (PCs), a procedure that allowed us to retain between 50% and 78% of the total variance (63% on average). Computation of the PCs was performed using the ‘prcomp’ function within the statistical programming environment R (script available on request).

### Inference of biological networks linking lung and skeletal muscles in guinea pigs

An exhaustive list of genes annotated to the cytokine superfamily (n = 72) was compiled from the SABiosciences PCR Array Web Portal [31] (see Additional file 5, worksheet 1 for the complete list). Such an approach has been used previously for compiling gene-lists [32]. Among these candidates we identified 33 genes coding for cytokines, which were differentially expressed in guinea pig lung tissue (Additional file 5, worksheet 2). These were selected for further analysis. Correlation between the expression of these cytokines and skeletal muscle pathway indexes were computed using the Spearman correlation coefficient, which allows the identification of linear and non-linear monotone relationships [33]. Resampling of samples (10,000 permutations) was conducted to obtain *P* values for each correlation coefficient. Pair-wise associations within the regulatory network were defined as statistically significant when *P* <0.01.

The resulting sparse network was visualised using a force-directed layout as implemented in the network visualization tool Cytoscape v2.8 [34].

### Creating and visualising a KEGG pathway map

To visually represent the relationship between enriched KEGG pathways in the clinical dataset, we computed a pathway similarity matrix based on the Jaccards Index of overlap. This matrix was used as input to a hierarchical clustering procedure (average linkage).

### Measurement of inflammatory mediators in human serum from COPD patients and healthy controls and validation of the guinea pig lung-muscle cross-talk network

Previously published multiplex protein profiling data from COPD serum samples (n = 26) and healthy controls (n = 23) were included [26]. Briefly, data were log2 transformed followed by imputation of missing values using K nearest neighbours in the R package ‘impute’ [35]. Finally, the full data matrix was Z-scored.

A Mack-Skillings test with two factors (disease and training) was used to identify overall main effects across groups in serum protein levels (*P* <0.05). A Gene Set Enrichment Analysis (GSEA) was used to establish statistical functional enrichment by ranking all Pearson correlation coefficients between serum protein levels and global muscle mRNA expression [36].

## Results

### Sequencing of the guinea pig transcriptome and development of a genome-wide guinea pig microarray platform

Illumina RNA sequencing (RNAseq) in this study has facilitated the construction of a comprehensive transcriptome for guinea pig lung and skeletal muscles, with much higher coverage than attainable purely by public available data. In combination with public domain data, Ensemble cDNAs and Genscan gene predictions, we have generated the first comprehensive annotation of the genome-wide transcriptome consisting of 151,072 transcript sequences (of which 81,074 were derived solely from the RNAseq data). The number of transcripts annotated with a RefSeq sequence, by stringent BLAST searching against mouse transcripts from NCBI’s RefSeq collection, was 97,822. This represented 17,907 non-redundant mouse gene symbols. Annotated genes were classified according to GO categories: cellular component (CC), biological process (BP) and molecular function (MF). Additional file 1: Figure S1 and S2 depict the distribution of the major GO categories at level 1; for comparison we also included level 1 GO terms for the mouse transcriptome. Overall, the guinea pig GO term representation is very comparable to that of the genes annotated in the full mouse genome, highlighting the generality of the assembled guinea pig transcriptome (Additional file 1: Figure S1 and S2). Only reproduction processes and extracellular region are poorly represented in the guinea pig transcriptome. In addition, by Human ortholog identification, it was shown that the guinea pig transcriptome assembled in this work contained genes included in the entire set of Human KEGG pathways available for download via the Broad Institute’s MSigDB Collections (see ‘KeggGPandMouseCounts.xlsx’ associated to Additional file 1).

Using the transcript assembly we have developed the first genome-wide microarray platform for the guinea pig model species. Based on the probe performance using an initial 180 K, we developed a 60 K custom Agilent microarray, representing 17,896 unique genes. Importantly, we are able to demonstrate a high concordance between our custom 60 K array platform and RNAseq data (Additional file 1: Figures S5, S6 and S7), particularly when the ratio between gene expression in lung and muscle tissue is compared (Additional file 1: Figures S8 and S9).

### Chronic exposure to smoking and/or hypoxia induces transcriptional changes in both lung and skeletal muscle in guinea pigs

**Figure 1:**
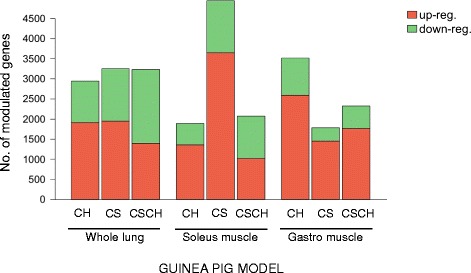
**Differentially expressed genes in the guinea pig experimental model.** Barplot summarising the number of differentially expressed genes identified by SAM analysis (FDR <1%) following long-term exposure to hypoxia (CH), chronic smoking (CS) or smoking followed by hypoxia (CS + CH) in whole lung and skeletal muscles (soleus and gastrocnemius).

To access whether the current guinea pig model show a transcriptional response, we used our newly developed microarray platform (Additional file 1) with mRNA extracted from whole lung and two metabolically distinct hindlimb muscles (*gastro* and soleus) sampled from shams and nose-only CS exposed animals approximately 1 month after a significant (*P* <0.01) decrease in body mass gain could first be detected [15]. Indeed, we were able to identify a relatively large number of genes differentially expressed in all three tissues from all experimental groups (Figure 1; see Additional file 6 for individual gene changes). Notably, both hindlimb muscles examined displayed a marked response, with the oxidative soleus muscle showing the largest number of changes following exposure to CS alone (Figure 1).

### Lung response to the different stressors is comparable in magnitude, but involves different subsets of functional pathways

**Figure 2:**
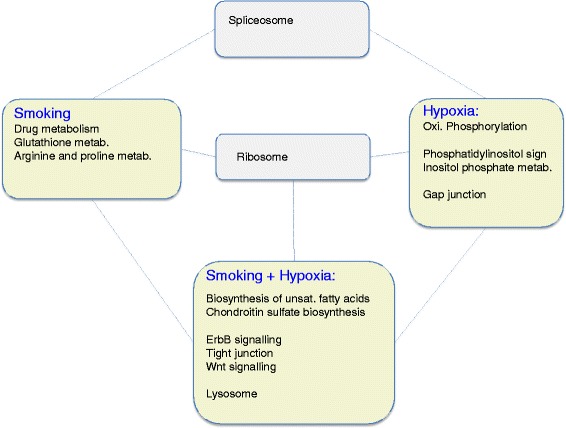
**Overlap analysis of enriched KEGG pathways in guinea pig lung tissue.** Rectangles coloured in green indicate pathways unique to a specific experimental condition (that is, CS, CH or CSCH). Grey-coloured rectangles indicate pathways that are regulated by more than one condition.

Having shown that both lung and muscles mount a quantitatively comparable transcriptional response following the different experimental challenges, we then compared and characterised such responses at the functional pathway level. We first focused on lung tissue and performed a functional enrichment analysis, which identified 15 KEGG pathways (see Additional file 7 for the result of the GO analysis) that were enriched in genes differentially expressed in lungs, in at least one experimental condition (Figure 2). Only genes annotated to the ribosome pathway were significantly modulated by all three experimental conditions. Instead the vast majority of differentially modulated pathways were equally distributed between the specific exposure conditions. The six pathways (40%) unique to the CSCH group could be grouped into two main functional categories: (1) biosynthetic pathways, and (2) pathways with a strong signalling component such as ErbB- and Wnt signalling as well as tight junction. In contrast, all three pathways (that is, cytochrome P450 drug-, glutathione-, and arginine and proline metabolism) enriched in genes upregulated by long-term smoking *per se* could be associated to metabolic processes primarily involved in detoxification of oxidative stress. Finally, the four enriched terms specific to hypoxia can be broken down into two main functional classes: (1) an oxygen-dependent bioenergetic component (Oxidative Phosphorylation) enriched in genes downregulated by hypoxia, and (2) pathways with a strong signalling component also negatively affected by hypoxia (that is, phosphatidylinositol signalling, inositol phosphate metabolism and gap junction).

### Glycolytic and oxidative limb muscles respond differently to either smoking or hypoxia

**Figure 3:**
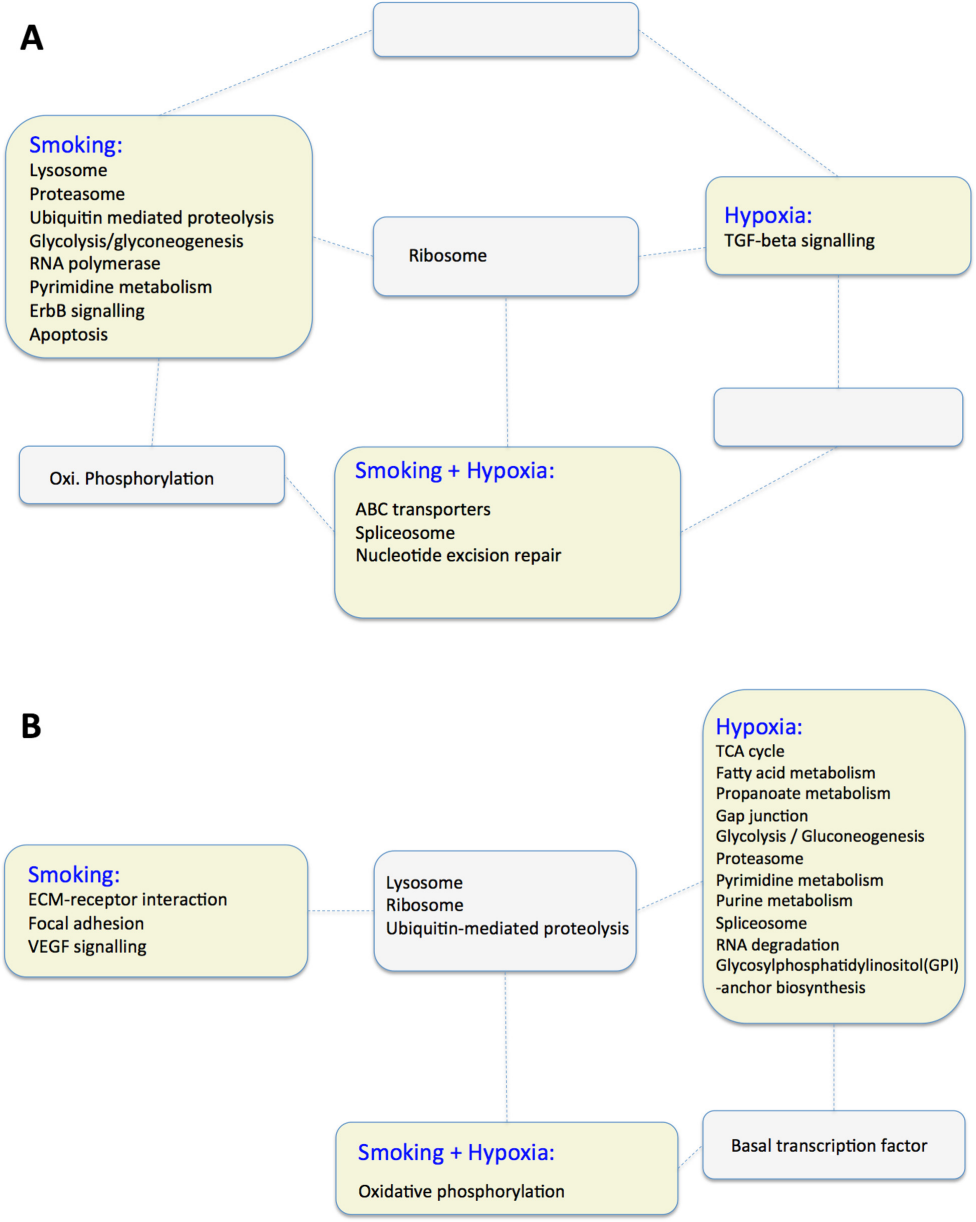
**Overlap analysis of enriched KEGG pathways in two guinea pig hindlimb muscles with discrete metabolic profiles.** Rectangles coloured in green indicate pathways unique to a specific experimental condition (that is, CS, CH or CSCH). Grey-coloured rectangles indicate pathways that are regulated by more than one condition. **(A)** Soleus muscle; **(B)** gastrocnemius muscle.

The lateral gastrocnemius modulated a larger number of genes following hypoxia, whereas the soleus (an oxidative muscle) responded preferentially to the smoking challenge (Figure 1). This trend was even more evident when testing for functional pathway enrichment. The soleus muscle responded to CS exposure by modulating 10 of the 13 pathways (77%) identified as differentially regulated in at least one sample group, making this muscle the most sensitive to this stressor (Figure 3A). Of these, eight were specifically modulated by CS.

The response to hypoxia only involved two pathways of which one was specific to CH (TGF-beta signalling). However, hypoxia has a considerable effect in the CSCH exposure, effectively reducing the impact of CS (only five pathways were enriched when both stimuli were combined).

Consistent with the gene-level analysis, the gastrocnemius muscle primarily showed functional pathway enrichment in response to hypoxia (Figure 3B). Genes differentially regulated in this condition, were enriched in 15 of the 19 KEGG pathways (79%) modulated in this muscle in at least one of the experimental challenges. These could be grouped in to three main functional clusters: (1) bioenergetic pathways such as glycolysis and TCA cycle, (2) metabolic pathways (for example, fatty acid-, propanoate-, purine- and pyrimidine metabolism), and (3) pathways exerting degradative processes such as proteasome and ubiquitin-mediated proteolysis. Only three pathways, all with a tissue-remodelling component, were specific to the CS group (focal adhesion, VEGF- and ECM-receptor signalling; Figure 3B). As a further contrast to the oxidative soleus muscle (Figure 3A), aerobic energy metabolism *in gastro*, represented by OxPhos, was only enriched among downregulated genes when hypoxia was added on top of the CS challenge.

### The guinea pig smoking model recapitulates the transcriptional changes observed in human COPD skeletal muscles

Having described the pulmonary as well as extrapulmonary transcriptional response to long-term CS and/or hypoxia, we assessed their clinical relevance with an initial primary focus on peripheral skeletal muscle. This was achieved by comparing the enriched transcription-based functional profiles derived from the guinea pig model with the functional profile of genes differentially expressed in quadriceps muscle biopsies from COPD patients relative to matched controls (see Additional file 4 for the list of regulated transcripts as well as functional enrichment analysis) [37].

**Figure 4:**
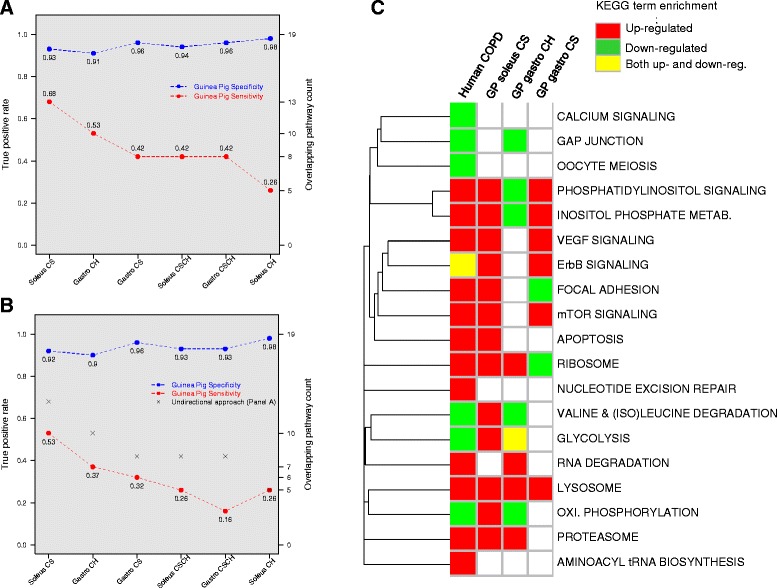
**Muscle specific pathway-level comparisons between the guinea pig model and a clinical COPD study (GSE27536).** The figure displays the result of the sensitivity and specificity analysis for each of the six experimental conditions at the KEGG pathway level, where ‘true response’ is defined by the 19 enriched KEGG pathways in the *vastus lateralis* of muscle-wasted COPD patients when compared to matched healthy controls. **(A)** Plot where the x-axis represents the experimental conditions in the guinea pig model. The two y-axes represent (1) the true positive rate as a fraction, and (2) the actual number of overlapping pathways with the human COPD cohort. In this panel specificity and sensitivity is computed without considering the direction of change in expression (up- and downregulation). **(B)** The equivalent of the plot in (A) where specificity and sensitivity is computed taking into consideration the direction of change. The grey-coloured crosses indicate the sensitivity values from the undirected approach presented in (A). **(C)** represents the specific pathways regulated in the guinea pig model (columns 2 to 4) when contrasted against COPD patients with a muscle-wasting phenotype (column 1). Green-coloured cells indicate enrichment among downregulated transcripts; red-coloured cells indicate enrichment among the upregulated transcripts; and yellow-coloured cells indicate enrichment for transcripts enriched in both directions.

First, we defined the transcriptional signature representing muscle wasting in human COPD by comparing a cohort of COPD patients with low FFMI to matched healthy individuals. This identified 1,861 differentially regulated genes (1,416 up- and 445 downregulated), which represented 19 unique functionally enriched KEGG pathways (Figure 4C). In order to assess which experimental challenge best mirrored human COPD, we performed a sensitivity and specificity analysis where ‘true response’ was defined by the 19 KEGG terms (KEGG group 1-4) representing dysfunction human COPD muscle. This analysis, performed ignoring the direction of change, showed that a large percentage of enriched KEGG terms in guinea pigs did overlap with the COPD pathway signature - irrespective of the exposure (specificity scores ranging from 91% to 98%, *P* <0.01) (Figure 4A). Soleus muscle derived from CS-exposed guinea pig showed the highest sensitivity, with 13 out of 19 (68%) KEGG pathways in common (Figure 4A; additionally regulated genes have been mapped to key pathways (Additional file 8)). The *gastro* muscle from hypoxic guinea pigs followed by a short measure with a sensitivity of 53% and 10 KEGG pathways in common. The pathway overlap was statistically significant in all six experimental conditions (*P* <0.01).

The same comparison, this time only considering those KEGG pathways that were enriched in genes with the same direction of regulation as the human dataset (Figure 4B), still revealed a significant overlap with the human dataset for five out of six experimental challenges (only CSCH-exposed gastro had a *P* >0.01). On average the sensitivity only decreased by 14% (Figure 4B) indicating that most of the response is in the same direction.

We conclude that the transcriptional response of guinea pig limb muscle to long-term CS accurately reflects the transcriptional state of dysfunctional limb muscles in COPD patients. From a biological standpoint, many of the KEGG pathways in common between the guinea pig model and human COPD relate to (Figure 4C):

1) increased tissue remodelling (VEGF signalling, focal adhesion, apoptosis)

2) altered energy metabolism (oxidative phosphorylation, glycolysis)

3) increased proteolysis

Thus, we demonstrate the validity of the guinea pig smoking model as the basis for further mechanistic investigation, to facilitate better clinical therapy.

Noteworthy, a similar approach comparing guinea pig and human lungs from healthy and COPD smokers highlighted a less striking, albeit significant response (*P* <0.05) (Additional file 3: Figures B and C). Of the 2,584 genes regulated in small airway epithelium from smokers with COPD compared to healthy non-smokers (832 up- and 1,752 downregulated), which represented 20 unique functionally enriched KEGG pathways (Additional file 4 - worksheets 3 and 4), only six pathways (30%) were in common with the CSCH group in our guinea pig model. Whereas this animal model does capture the CS-induced oxidative stress response (that is glutathione metabolism and metabolism of xenobiotics by cytochrome P450 are both induced), our functional gene-level comparison indicates a ‘metabolic gap’ in the current guinea pig smoking model, which is particularly pronounced for amino acid metabolism (Additional file 3: Figure B).

A significant fraction (60%) of the enriched pathways in smokers with COPD are also modulated in healthy smokers without COPD. Of the pathways regulated in healthy smokers compared to non-smokers, three pathways (25%) overlapped with the smoking guinea pig model (that is, glutathione metabolism, pentose phosphate- and the proteasome pathways) (Additional file 3: Figure B).

### Gene expression of lung soluble inflammatory mediators correlate with skeletal muscle gene expression

**Figure 5:**
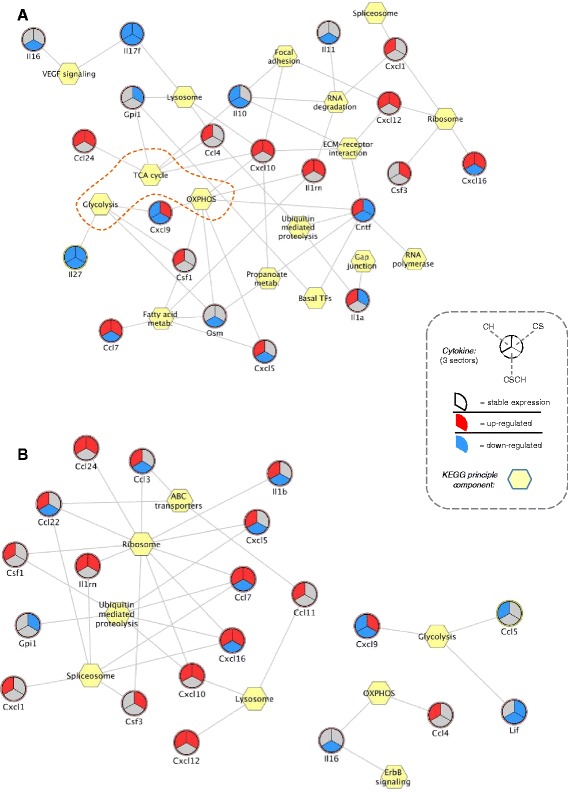
**Networks representing the transcriptional coupling between lung and skeletal muscle in the guinea pig smoking model.** Correlation network linking the expression of soluble factors in guinea pig lung with the transcriptional activity of enriched KEGG pathways in gastrocnemius **(A)** and soleus **(B)** muscles, respectively. Each network edge (grey line) represents the correlation with a given principal component, representative of several genes in each function/KEGG term. Some of the genes will be correlated positively and others will show a negative correlation. Only edges with a bootstrapped *P* value <0.01 are shown in this figure. Hexagons represent KEGG terms, and circles represent soluble factors that are secreted by lungs. Each circle has been divided into three sectors, representing the CS, CSCH and CH groups. A red sector indicates that the cytokine is significantly upregulated; a blue sector indicates significant downregulation; and a grey-coloured sector indicates that the transcriptional level is not significantly affected by the experimental condition.

Having demonstrated that the transcriptional state of guinea pig hindlimb muscles following CS exposure represents that of dysfunctional COPD muscle well, we then determined whether we could reverse engineer [33] the structure of a gene regulatory network, linking gene expression of lung soluble factors to pathway indices representing the muscle transcriptional state. Thirty-three of the 72 genes annotated to the cytokine superfamily (46%, Additional file 5) were differentially expressed in at least one of the experimental groups and were selected for reverse engineering. We next computed the Spearman correlation coefficients between the profile of expression of each factor in lung and indexes of KEGG pathway activity in skeletal muscles. After removing correlations with *P* >0.01, the union of the soluble factors neighbourhoods was visualised using a force-driven layout (Figure 5).

Analysis of the *gastro* network (Figure 5A) revealed two cytokines each with six edges (that is, Cntf and Cxcl10), indicating that these hub genes could exert an effect on multiple pathways within the network. The topological analysis also revealed a dense connected area within the network that was enriched of energy metabolism pathways (OxPhos, TCA cycle and glycolysis). Interestingly, Cxcl10, whose expression level was significantly increased in both the CSCH and CH groups (observed with both microarray and qPCR - see Additional file 9), was connected to both members within this energy metabolism dense area of the network that comprised aerobic respiration (that is, OxPhos and TCA cycle). Noteworthy, Cxcl9, which targets the same receptor as Cxcl10, also linked to both oxidative phosphorylation and glycolysis.

Visual inspection of the soleus network (Figure 5B) revealed that Cxcl9 was still linked to bioenergetic processes via the glycolysis pathway. Notably, Cxcl10 was now unconnected to energy metabolism pathways. Instead, this cytokine among others linked to the ribosome component within the network, which had the highest number of connections (11 edges) (Figure 5B).

We may thus hypothesise that some of the cytokines we have identified in the guinea pig may also act as systemic signals in COPD patients and be responsible for reducing energy provision in skeletal muscle.

### Serum cytokine profiling in human COPD patients confirms the predictions of the guinea pig model

Having shown a remarkable similarity between the transcriptional state of guinea pig and human COPD muscles, we next assessed whether the link between expression of selected pulmonary cytokines and the transcriptional activity of enriched KEGG pathways in peripheral guinea pig muscles was of clinical relevance. For this analysis we took advantage of relevant measures from a COPD serum profiling dataset (that is, CXCL9, CXCL10, CCL4, CCL5, CCL11, IL1beta and VEGF) used in a previous publication from our group [26] in order to test whether (1) serum cytokine protein levels were affected by disease and/or prolonged endurance training, and (2) if they were correlated with skeletal muscle gene expression. Of the seven cytokines included, we could indeed verify that the serum protein level of both CXCL9 and CXCL10 were significantly modulated in COPD patients, irrespective of their FFMI, compared to healthy controls (Additional file 10). More specifically, CXCL10 human protein levels were higher in COPD patients. This increase was consistent with the observed up regulation of mRNA in the guinea pig lung. However, the increase in CXCL9 human protein levels only fit with the increase in mRNA levels in guinea pig lungs exposed to CH.

### Training did not modulate any of the tested cytokines

**Figure 6:**
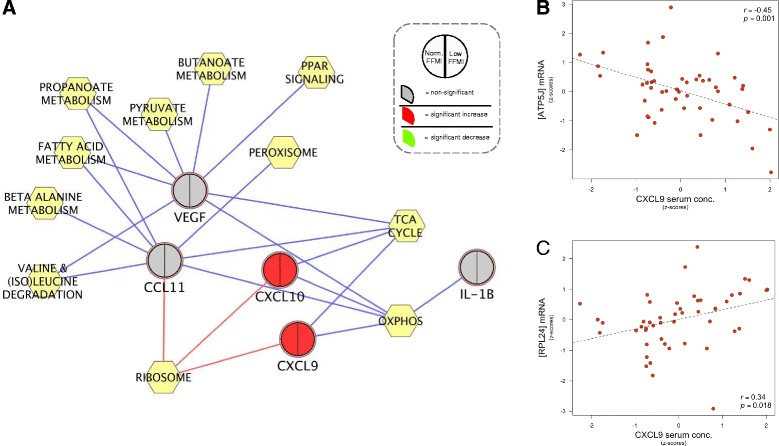
**Network representing the correlation between serum cytokine levels and skeletal muscles gene expression in the human COPD study. (A)** The force-directed network representation linking the serum level of selected chemokines/cytokines (round nodes) with enriched KEGG pathways (yellow hexagons). Each circular node has been divided into two sectors, representing COPD patients with (1) a normal FFMI and (2) a low FFMI. A red sector indicates that the cytokine level is significantly higher compared to matched controls, and a grey-coloured sector indicates that the serum protein level is stable between groups. Blue edges mean negative correlation, whereas red edges mean positive correlation. **(B, C)** Scatterplots representing the association between serum levels of CXCL9 (x-axis) and muscle mRNA expression (y-axis) of examples of genes involved in oxidative phosphorylation (B) and ribosomal biogenesis (C).

We next correlated all mRNA transcripts expressed above background (12,783 genes) in skeletal muscle in the same cohort with serum cytokine levels and tested whether specific KEGG pathways were significantly enriched between the positively or negatively correlated genes. Encouragingly, these results were remarkably similar to the guinea pig correlation networks shown in Figure 5. The similarity was particularly evident in respect to the inverse correlation between CXCL9 and CXCL10 serum protein levels and the expression of aerobic energy metabolism genes in muscle (OxPhos and TCA cycle) (Figure 6). Importantly, these strong negative associations to aerobic energy metabolism genes were still present if we ignored the control samples, demonstrating that VO_2_max difference is not a main component of the correlations (Additional file 11).

## Discussion

The most important finding of this study is the discovery that mRNA levels in CS-exposed guinea pig lungs, as well as human serum protein levels of CXCL9 and CXCL10, are significantly inversely correlated to the expression of aerobic energy metabolism genes in skeletal muscles. In addition, we demonstrate that the guinea pig smoking model can mimic many of the transcriptional changes observed in limb muscle of atrophic COPD patients, making this an extremely useful *in vivo* experimental model.

### The role of systemic inflammatory cytokines in controlling the molecular state of skeletal muscles

Several human studies have demonstrated that COPD is associated not only to inflammation of the lungs, but also with increased levels of circulating pro-inflammatory cytokines [38,39]. Notably, there is a clear trend toward significant induction of TNF-*α* plasma levels of CS-exposed guinea pigs, suggestive of a similar systemic inflammatory process in this model organism [16]. Although elevated levels of pro-inflammatory cytokines have previously been linked to skeletal muscle dysfunction [40], until now it was unclear whether they are the primary factor driving muscle wasting.

The analyses we have performed provide the first evidence that expression of systemic cytokines significantly correlates with expression of energy metabolism genes (represented by the OxPhos, TCA and glycolytic pathway) in limb muscles (Figures 5 and 6). This is consistent with the previous observation that dysfunctional limb muscle of COPD patients are unable to co-ordinate the expression of energy metabolism genes [26].

Importantly, there is strong evidence that most of the candidates we have identified in the guinea pig model are also modulated in COPD patients. For example, it has previously been shown that serum levels of CCL24, CSF1 and IL1A (among others) are significantly higher in COPD patients compared to matched controls [41]. Our own validation of selected model predictions demonstrated that serum protein levels of two CXCR3 chemokines (CXCL9 and CXCL10) are significantly higher in the same COPD cohort used for muscle mRNA profiling, and their levels negatively correlate with the expression of energy metabolism genes in human COPD skeletal muscle (see Figure 6A). Consistent with this observation (see also [42]) we found a statistically significant negative association between CXCL9 and CXCL10 serum protein levels and the distance walked in 6 min (Additional file 12). Noteworthy, exercise training did not modulate serum levels of these chemokines, indicating that differences in the level of physical activity overall will not affect serum levels of these chronic inflammatory mediators.

Overall, this strongly suggests that systemic inflammation plays a major role in promoting skeletal muscles dysfunction in both smoking guinea pigs and human COPD patients.

### Biological significance of the transcriptional response to smoking and/or hypoxia in lungs and muscles

In accordance with the (pre-)clinical literature, we identified a massive transcriptional response in whole lung tissue (Figure 1). As anticipated, the result of the functional enrichment analysis suggests that long-term CS exposure induces pathways related to the antioxidant defence system (that is, glutathione and cytochrome P450 drug metabolism), most likely in order to try and cope with the increased ROS production. Such responses are consistent with the changes observed in the human airway transcriptome of chronic smokers [43,44] (Additional file 3).

Interestingly, the two skeletal muscles showed a distinct pattern of response both at the gene and pathway level, likely reflecting differences in fibre type composition, and hence metabolic profile, between these two hindlimb muscles. Soleus is a postural, entirely oxidative muscle, with a high Type I fibre content, whereas the gastrocnemius is a phasic muscle of mixed Type II composition that is predominantly glycolytic.

Our data suggest that CS *per se* primarily affects the soleus (Figure 1) similar to a recent finding in C57 mice, where contractile properties were selectively affected in soleus following chronic CS exposure using a nose-only device [12]. Furthermore, only the soleus showed a tendency towards lower muscle mass after mice had been whole-body exposed to CS for 6 months [11].

The result of the pathway level analysis in guinea pig soleus clearly suggests that long-term smoking modulates the activity of the MAPK pathway as induced signalling pathways (that is, ErbB signalling and apoptosis) represent components of this pathway. This agrees with a recent human study in which several key members of the MAPK pathway were upregulated at the mRNA level in *vastus lateralis* of patients with COPD [45]. In addition, aerobic energy metabolism is also clearly hit in the CS-group, which is also consistent with findings in skeletal muscle of human COPD patients [46].

One puzzling finding in the current study is that hypoxia appears to exert a ‘protective’ transcriptional effect in the soleus, as only a few enriched pathways were found when the hypoxic challenge was added to the CS intervention. A density plot of fold-changes revealed that CS as a single factor exerts a greater transcriptional effect than either of the two other experimental conditions where CH is present, among the transcripts with an absolute fold-change above 1.4. The reason for this is not clear and highlights the need for further research in this area.

In addition to the demonstrated agreement with published human transcriptional data, we also addressed the clinical relevance of the present animal model by comparing the transcription-based functional profiles related to each experimental challenge with the functional signature derived from stable yet severe COPD patients when compared to healthy age-matched controls. In accordance with results from our gene-level analysis and phenotypic data from the mouse smoking model, the response of the soleus to long-term smoke exposure best mimics expression signatures linked to the effects of COPD in human limb muscle, with 68% KEGG terms in common (Figure 4A). However, exposure to CH in *gastro* also yielded a highly statistically significant overlap with the clinical dataset as highlighted by a 53% functional overlap.

### Gene expression profiling as a tool to assess animal model relevance of human disease

Most pre-clinical models of CS-induced COPD have for obvious reasons focused on the lung component of the disease [47]. However, with the increasing awareness of the clinically important extrapulmonary manifestations linked to COPD, a number of studies have now begun to try to elucidate the mechanisms governing skeletal muscle dysfunction, whether or not accompanied by loss of muscle mass. Consistent with COPD, previous studies have shown marked reduction in body weight gain [15,16] as well as increased oxidative stress [6] in guinea pig hindlimb muscles following only 3 months of daily CS exposure. In contrast, macroscopic data suggest that mouse models poorly reproduce the systemic effects of human COPD. For example, long-term exposure to CS only induces mild effects in selected skeletal muscles, as defined by fibre redistribution and altered oxidative enzyme activity [11,12], despite both studies using much longer exposure protocols than that of the present study.

It may be important to assess and compare the relevance of the current guinea pig model with that of other rodent CS models such as the mouse. Although an extensive microarray analysis of such model has not been published, we have been able to retrieve a dataset from the GEO database (GSE18033). In order to comment on the suitability of this dataset to address this important issue, we analysed this data using the same approach described in this paper (see Additional file 13 for the full analysis).

The experiment performed was limited to the gastrocnemius, which our analysis in the guinea pigs suggests is not the most representative of human COPD. However, before any comparison could be made, our analysis only identified 24 genes that were differentially modulated by chronic CS-exposure (24 weeks) in hindlimb gastrocnemius muscle compared with time-matched sham controls at a reasonable statistical threshold (FDR <15%). Only by raising the statistical cutoff to 30%, which increased the number of regulated transcripts to 1,020 (Additional file 13: Table S3), could we detect biologically relevant functions, although the maximum sensitivity of 0.11 was substantially lower than any of the experimental conditions involving CS-exposure in the current guinea pig model (Figure 4A). Although further analysis of the mouse model is required for reaching any definitive conclusion, these results indicate poor transcriptional response following smoking exposure in mice.

## Conclusions

There are a number of indications that the current nose-only guinea pig smoking model is useful for studying extrapulmonary effects of COPD, as previously pointed out, including an oxidative stress phenotype in limb muscle as well as induction of plasma inflammatory mediators.

The data presented herein provide further evidence for the utility of the guinea pig smoking model for replicating key clinical traits in human COPD, particularly as a tool for identifying systemic signals influencing peripheral skeletal muscle function. Hence, these data should facilitate more detailed interrogation of current guinea pig models of human disease, likely promoting further therapeutic development.

## Additional files

Additional file 1
**In depth description of mRNA sequencing analysis and development of a custom microarray platform** [20,21,30,36,48-69 **].**
Additional file 2
**Table with physiological characteristics of the clinical COPD cohort.** Data are presented as means ± SEM. Additional file 3
**Analysis of the genome-wide transcriptional response in lung tissue from COPD patients, healthy smokers and healthy non-smokers, respectively (GSE19407).**
Additional file 4
**Result of the SAMR differential gene expression analysis and KEGG pathway enrichment analysis in the two human COPD cohorts (GSE27536 and GSE19407) and the public available mouse smoking dataset (GSE18033; presented in Additional file** 13 **), respectively.**
Additional file 5
**List of genes that are (1) annotated to the cytokine superfamily (n = 72) and (2) differentially expressed in whole lung tissue of treated guinea pigs compared to untreated controls (n = 33; FDR <1%).**
Additional file 6
**Result of the SAMR differential gene expression analysis in the guinea pig smoking model for each of the experimental conditions the lung and muscle, respectively.**
Additional file 7
**Lists of statistically enriched Gene Ontology terms (FDR <5%) in the different experimental conditions in guinea pig lung (A) and hindlimb muscles (soleus: B;** ***gastro*** **: C), respectively.** Numbers in red squared brackets indicate the specific cluster number from the output of DAVID. The black open brackets give the number of enriched genes for a given ontology term. Additional file 8
**KEGG pathway diagrams for five key pathways that are functionally enriched in soleus muscle of guinea pigs exposed to long-term CS.** Each gene in a pathway has been divided into four sectors in order to demonstrate how that gene is regulated in the guinea pig model as well as in the limb muscle of COPD patients when compared to their respective controls. Green indicates downregulation; red indicates upregulation. Additional file 9
**CXCL9 (A) and CXCL10 (B) mRNA levels in lung tissue of sham controls (CON), cigarette smoke-exposed (CS), hypoxic (CH) and to combined stimuli (CSCH).** Values (means ± SE (n = 3-4 guinea pig/group)) in the treated groups are presented as relative to the untreated sham controls (basal level = 1). * Significantly different from the control group (*P* <0.05). (*) tends to be significantly different among treated (0.05 > *P* <0.1). Additional file 10
**Heatmap visualising the serum protein levels of selected cytokines in the human COPD cohort with the extrapulmonary focus (GSE27536).** Each row represents a cytokine, whereas each column represents a human subject. Red colours mean increased expression whereas green colours mean decreased expression. An asterix denotes significance at *P* <0.05 for the disease factor (Mack-Skillings test). Additional file 11
**Scatterplots highlighting that the negative associations between CXCL9/-10 serum protein levels and muscle aerobic energy metabolism genes in the human COPD cohort are independent of exercise tolerance.** Data have been standardised (z-scored). Additional file 12
**Scatterplots relating the distance walked in 6 min to the serum protein levels of CXCL9 and CXCL10, respectively.**
Additional file 13
**Analysis of the genome-wide transcriptional response of hindlimb skeletal muscle in C57 mice exposed to long-term nose-only cigarette smoke [** 24,70,71 **].**

## Abbreviations

BMI: Body mass index
CH: Chronic hypoxia
COPD: Chronic obstructive pulmonary disease
CS: Cigarette smoke
CSCH: Chronic smoking and hypoxia combined
FDR: False discovery rate
FFMI: Fat free mass index
GEO: Gene Expression Omnibus
KEGG: Kyoto Encyclopedia of Genes and Genomes
PC: Principal component
PCA: Principal component analysis
SAM: Significance Analysis of Microarray
