# Supplementary material for: A systems biology approach reveals a link between systemic cytokines and skeletal muscle energy metabolism in a rodent smoking model and human COPD
Source: Genome Med. 2014 Aug 9;6(8):59. doi: 10.1186/s13073-014-0059-5 (PMC4165371; doi:10.1186/s13073-014-0059-5)
Supplement: Additional file 1 — In depth description of mRNA sequencing analysis and development of a custom microarray platform [20,21,30,36,48-69 ]. [file 13073_2014_59_MOESM1_ESM.docx]

Additional File 1

RNA sequencing analysis and the development of a guinea pig microarray platform

Summary

So far, probably because of an incomplete genome sequence and consequently the lack of a genome-based microarray platform, genome-wide transcriptional profiling has not been used extensively in guinea pigs (*Cavia porcellus*). The first approximately genome-wide custom guinea pig microarray was very recently developed using a cross-species hybridisation technique [47].

Here, we describe the development of the first transcriptome-sequencing dataset for guinea pigs representing lung and skeletal muscles, as well as the development and validation of a novel genome-wide microarray platform.

Contents

1. Development of the guinea pig transcriptome
   1. RNAseq transcriptome assembly
   2. Combining RNAseq and public data
   3. New transcriptome statistics and annotation
   4. Viewing of transcripts and probes in the guinea pig genome
   5. Differential gene expression and a Gene Set Enrichment Analysis of the RNAseq data
   6. Grouping of transcripts and evidence of novel transcript/gene discovery
   7. Novel functional domains added to the transcriptome
   8. New representations of mouse orthologs from RNAseq transcripts
   9. New representations of mouse orthologs from RNAseq transcripts
2. Development of a 60K Custom Microarray Design

# Results

# 1.0 Developing the guinea pig transcriptome

Although guinea pig is a model species, at the time of writing there were only 464 genes found in the Reference sequence project database. To better understanding the transcriptional response of guinea pigs in response to smoking, a high coverage transcriptome was constructed using novel RNAseq data derived from lung and skeletal muscle tissues. This was combined with public domain data, Ensembl cDNAs and Genscan gene predictions. The transcript assembly was used to make a comprehensive custom guinea pig microarray for hypoxia and smoking analysis.

## 1.1 RNAseq transcriptome assembly

There is a genome sequence available for guinea pigs (<http://www.ensemble.org/Cavia_procellus/Info/Index>) and this was used in conjunction with the TopHat [20,48] and Cufflinks algorithms [21] to assemble transcripts based on genome alignments from the RNAseq data. The first stage was to align reads to the genome with TopHat, which requires the insert size of the RNAseq fragments to be known prior to mapping. This was determined using the SMALT alignment tool with the sampling option [49], aligning reads to publicly available transcript data. Table 1 lists the number of reads for each tissue and a summary of the fragment size distributions, which were verified using the final assembly.

## Table 1. Number of RNAseq reads and fragment sizes.

| Tissue | Paired end reads (n) | Length of reads, each pair | Fragment size | Fragment size standard deviation |
| --- | --- | --- | --- | --- |
| Muscle | 113,438,469 | 101 | 173 | 104 |
| Lung | 56,466,360 | 101 | 169 | 92 |

TopHat v1.3.3 was then used to align the paired-end reads of lung and muscle to the Cavia_porcellus.cavPor3.64 genome assembly, executed with the following options: insert size, closure-search, butterfly-search and coverage-search. The resulting TopHat genome binary alignment files were separately inputted to Cufflinks v1.1.0 to assemble tissue specific transcriptomes. The multiple read correction, upper quartile normalisation and fragment bias correction options were used. Lung and muscle transcriptomes were subsequently combined using the cuffcompare algorithm, which produced an RNAseq transcriptome consisting of 81,074 sequences. A control step was performed to ensure that maximum transcriptome coverage was attained. Unique exon positions from the cufflinks assembly GTF files were extracted and compared to the cuffcompare output. A number of sequences were not included in the cuffcompare output and these were added into the assembly.

## 1.2 Combining RNAseq and public data

A search on the NCBI Entrez system for the species *Cavia porcellus*, found 19,975 sequences [50]. The Cap3 software program [51] was used to cluster these public domain sequences and 1,762 contigs and 9,569 singletons were obtained. Added to these were the Ensembl GP cDNA and non-coding RNA sequences, from the download files Cavia_porcellus.cavPor3.64.cdna.all and Cavia_porcellus.cavPor3.64.ncrna.fa [52]. Finally, Ensembl Genscan predictions [53] were downloaded and compared to the RNAseq and public data with BLAST [54]. Any Genscan prediction with <= 99% coverage was added into the assembly. The final transcriptome to be used in array design consisted of 151,072 transcripts. Table 2 presents a breakdown of the different sources of transcripts.

## Table 2. A numerical breakdown of all the sequences.

| Source | Description | Total prior assembly | Post assembly total |
| --- | --- | --- | --- |
| RNAseq data | Cufflinks cuffcompare assembled transcriptome sequences from GP lung and muscle poly-A RNA | 81,074 | 81,074 |
| Cufflinks QC sequences | Cufflinks genome position check sequences | 1,871 | 1,871 |
| Ensembl all cDNA | a set of transcript sequences based on EST and other sequence alignment evidence; Cavia_porcellus.cavPor3.64.cdna.all.fa | 20,166 | 20,166 |
| Ensembl all ncRNA | A set of non-coding RNA from Ensembl; Cavia_porcellus.cavPor3.64.ncrna.fa | 5,963 | 5,963 |
| Ensembl Genscan | Ensembl abinitio Genscan gene predictions based on genome alignments | 53,615 | 30,667 |
| NCBI data | Public GP gene, mRNA and EST sequences, clustered with Cap3 | 19,975 | 11,331 |

## 1.3. New transcriptome statistics and annotation

A bullet point summary of the transcriptome statistics is provided below. This is an initial transcriptome that was used to design a custom 180K Agilent microarray. An N50 statistic provides a base pair sequence length (a weighted median value) where 50% of the entire transcriptome sequences are contained within this value, with lengths equal or greater than this value (www.broadinstitute.org/crd/wiki/index.php/N50). The N50 of the full transcript assembly was 3.2Kb.

- Total transcripts = 151,072
- Longest transcript = 53,613 bp
- Shortest transcript = 36 bp
- N50 statistic = 3,247 bp
- N90 statistic = 902 bp
- Mean size = 1,894 bp

Annotation of the full transcriptome prior to selection of microarray probes was performed using a BLAST search against the mouse Reference sequence project databases (Refseq) [55]. The number of transcripts annotated with a Refseq sequence were 97,822, consisting of 17,907 non-redundant mouse gene symbols. For the final 60K Agilent microarray design, a re-annotation was carried out due to the delay between full annotation and publication. This was done for the 86,044 transcripts that were represented on the final array, and the Refseq mouse data used was of August 2013. From the 80,044 transcripts of clusters that were mapped to the final array, 40,625 of them were annotated with a mouse gene. In terms of probes, 18,072 of the 60,984 probes on the final array were annotated with a mouse gene. This represents 17,676 non-redundant mouse genes. The Refseq mouse annotation of both the full transcriptome and the final array are accessible online: <http://pcwww.liv.ac.uk/~herberjm/Davidsen_et_al/Additional_file1.zip> (‘**Davidsen_et_al_Refseq_annotation.xlsx’**). Note that 235 probes match transcript clusters that hit more than one mouse gene (the probes matched all transcripts in a cluster but different transcripts of the cluster matched multiple mouse genes). These are highlighted in red in the ‘ProbeAnnotation’ sheet of the file **Davidsen_et_al_Refseq_annotation.xlsx** (also accessible online^[[1]](#footnote-1)^).


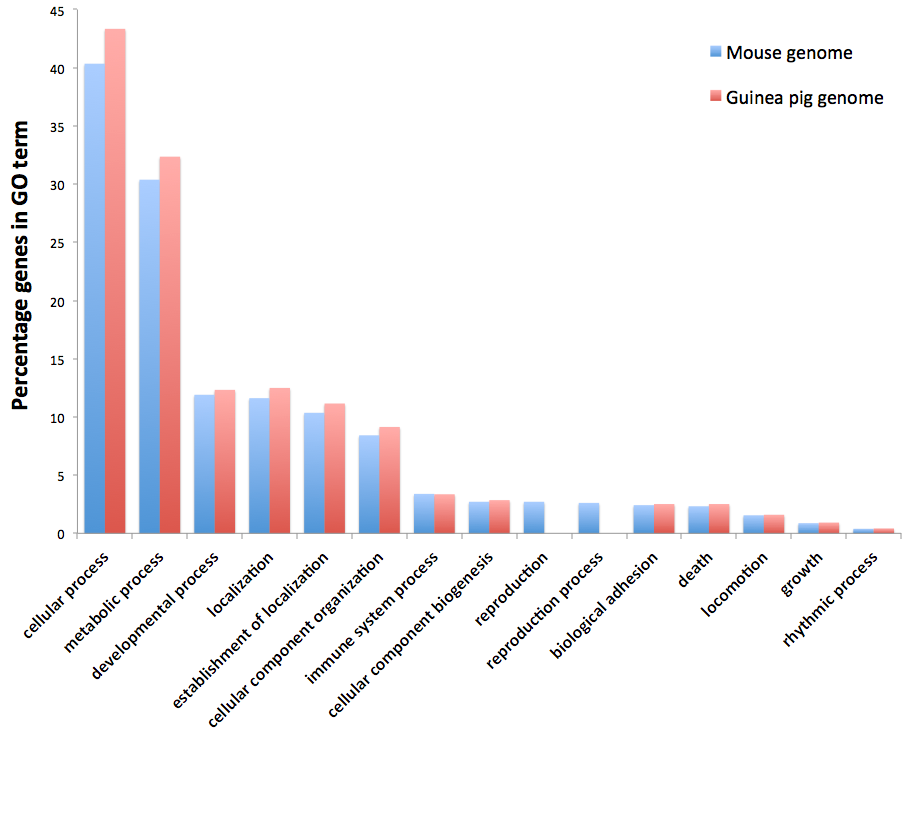


Figure S1. Barplot showing level 1 Gene Ontology (GO) terms of the biological process category. Blue bars represent all functionally annotated genes from the *Mus musculus* species whereas the red bars represent all annotated genes from the guinea pig RNAseq analysis.


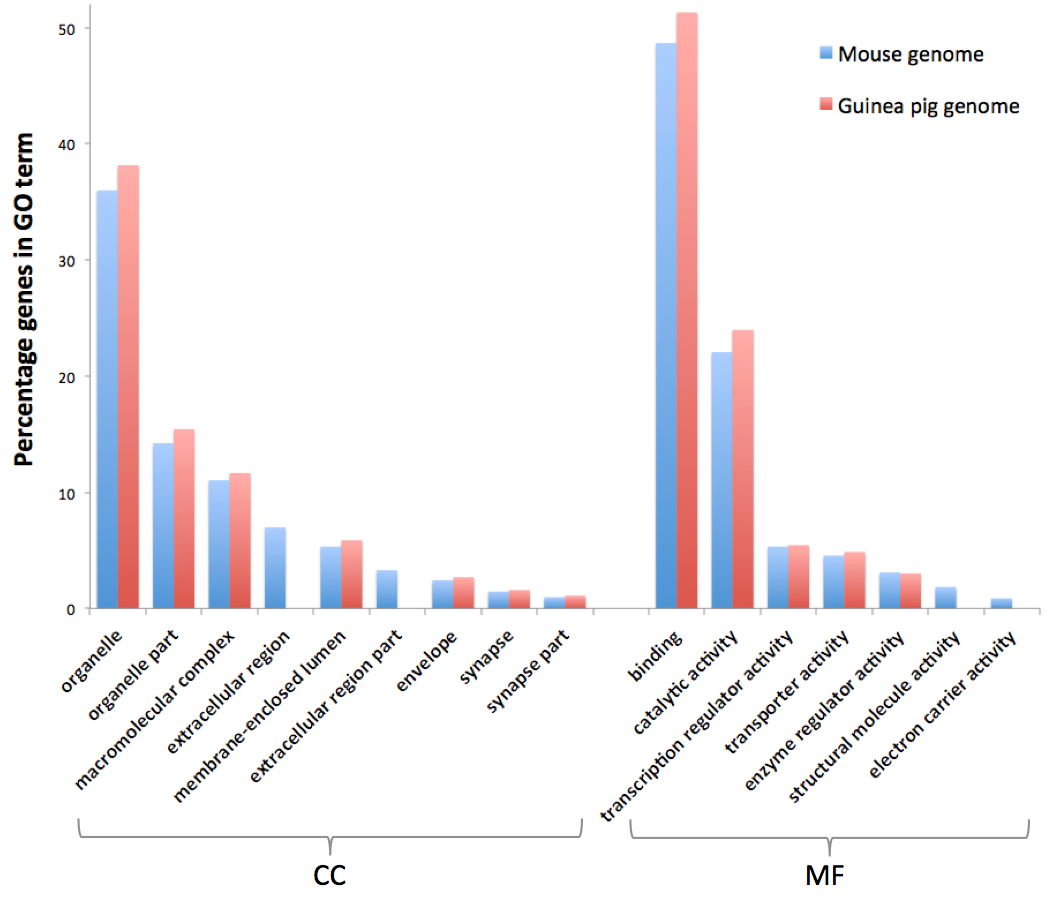


Figure S2. Barplot showing level 1 Gene Ontology (GO) terms of the cellular component (CC) and molecular function (MF) category, respectively. Blue bars represent all functionally annotated genes from the *Mus musculus* species whereas the red bars represent all annotated genes from the guinea pig RNAseq analysis.

## 1.4. Viewing of transcripts and probes in the guinea pig genome

A genome overview of both the guinea pig transcripts and microarray probes (see below for details on the development for the development of the microarray platform) has been provided and can be viewed via the University of California Santa Cruz genome browser [56,57]. This can be done by visiting the GP genome browser page at <http://genome.ucsc.edu/cgi-bin/hgTracks?db=cavPor3> and uploading the custom track file (**Davidsen_et_al_UCSC_genome_browser_custom_tracks.txt**) provided in the online supporting material^1^. Figure S3 shows an overview of the TMEM64 ortholog gene, with new transcripts displayed in red, and microarray probes in green (the top two tracks).


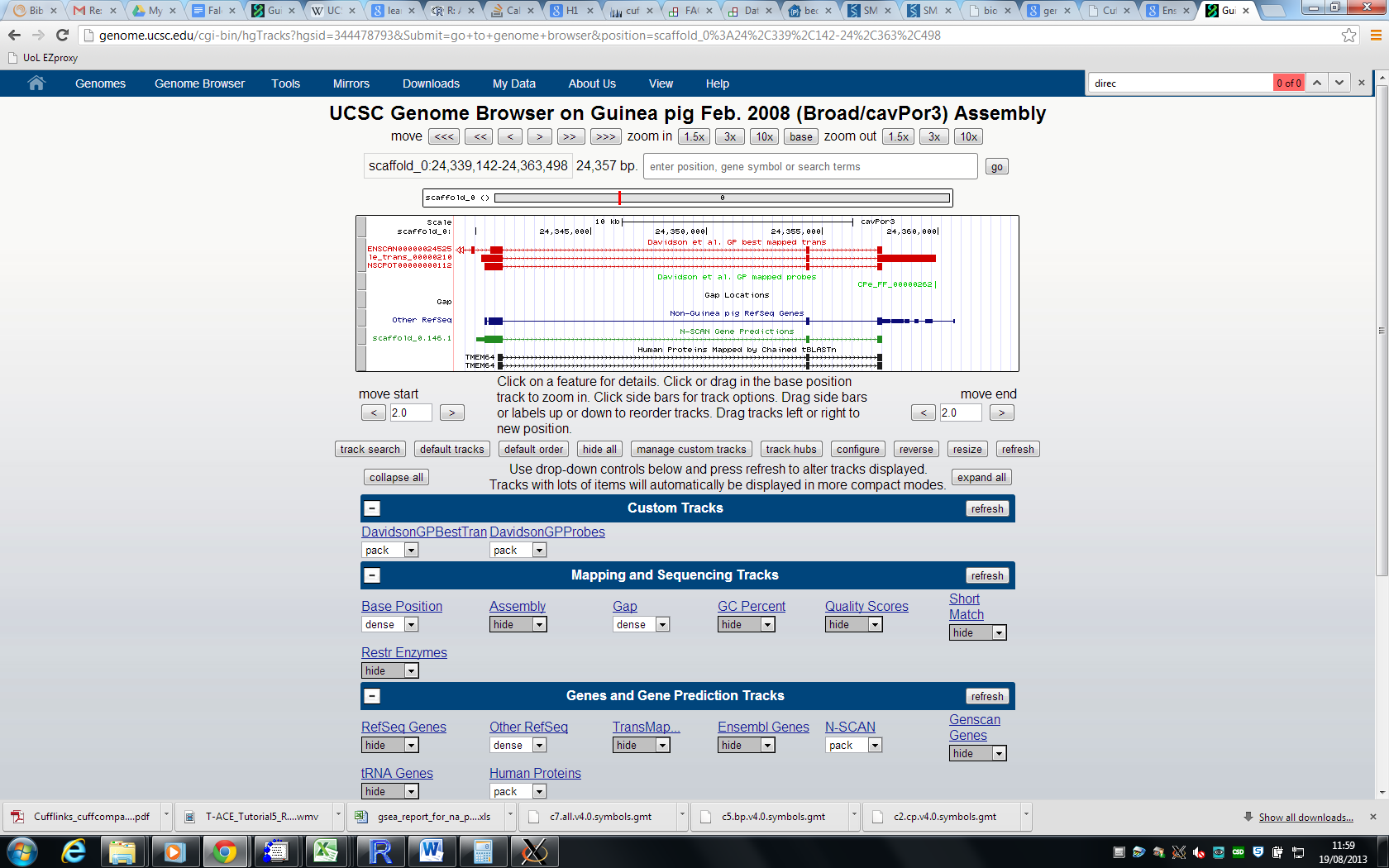


Figure S3

## 1.5. Differential gene expression and a Gen Set Enrichment Analysis of the RNAseq data

Single samples of lung and muscle were subject to RNAseq analysis to identify genes that distinguish between these two tissues. Because of the lack of biological replicates, transcript expression was contrasted using log2 fold-change between lung and muscle. To derive the fold-change, the level of expression was measured using the Fragments Per Kilobase of exon model, per Million fragments mapped (FPKM) to the genome, which is the metric calculated by the Cuffdiff program, from the Cufflinks package. This expression is based on genome alignments of RNAseq reads. For a comparison, expression was also measured using the eXpress package [58], which measures Reads Per Kilobase of exon model, per Million reads mapped. eXpress measures levels of reads mapping to the transcriptome and accounts for variation in fragment length distributions, read errors and fragment bias. The log2 fold-change measurements were used to make two ranked list of genes. The correlation of log2 fold-changes between the two methods of measuring RNAseq expression was 0.8. Transcripts were assigned mouse gene symbols based on similarity searching with BLAST.

A Gene Set Enrichment Analysis (GSEA) was carried out using the Broad Institutes command line java tool [35, 59]. This analysis was done using two genesets from the Molecular Signatures Database representing KEGG pathways [30, 60] and Gene Ontology biological processes [61]. The human orthologs of the mouse gene annotation was derived from Homologene [62]. For both KEGG and GO biological process, significant enrichment scores were found. Tables 3a and 3b display the most enriched ontologies and pathways for lung and muscle RNAseq data. Figure S4 overviews examples of the enrichment within the ranked lists, with lung enrichment on the left hand panels and muscle on the right. The results of the GSEA for both gene ontology and KEGG suggest that the muscle sample is enriched for muscle related processes such as muscle respiration, metabolism and contraction. Biological relevance is also found in lung enrichments, showing evidence of immune response, defence and inflammation reactions.

## Table 3A. Gene Set Enrichment Analysis of the RNAseq data (KEGG)

| Gene ontology biological process | Enrichment score | FDR | Tissue enriched |
| --- | --- | --- | --- |
| REACTOME_COMPLEMENT_CASCADE | 0.84 | 0 | Lung |
| REACTOME_INTERFERON_ALPHA_BETA_SIGNALING | 0.81 | 0 | Lung |
| REACTOME_CHEMOKINE_RECEPTORS_BIND_CHEMOKINES | 0.8 | 0 | Lung |
| KEGG_GRAFT_VERSUS_HOST_DISEASE | 0.79 | 0 | Lung |
| KEGG_AUTOIMMUNE_THYROID_DISEASE | 0.78 | 0 | Lung |
| KEGG_ALLOGRAFT_REJECTION | 0.78 | 0 | Lung |
| KEGG_COMPLEMENT_AND_COAGULATION_CASCADES | 0.75 | 0 | Lung |
| REACTOME_CYTOCHROME_P450_ARRANGED_BY_SUBSTRATE_TYPE | 0.74 | 0 | Lung |
| REACTOME_IMMUNOREGULATORY_INTERACTIONS_BETWEEN_A_LYMPHOID_AND_A_NON_LYMPHOID_CELL | 0.7 | 0 | Lung |
| REACTOME_INTERFERON_GAMMA_SIGNALING | 0.7 | 0 | Lung |
|  |  |  |  |
| REACTOME_CITRIC_ACID_CYCLE_TCA_CYCLE | -0.94 | 0 | Muscle |
| KEGG_CITRATE_CYCLE_TCA_CYCLE | -0.9 | 0 | Muscle |
| REACTOME_RESPIRATORY_ELECTRON_TRANSPORT | -0.89 | 0 | Muscle |
| REACTOME_RESPIRATORY_ELECTRON_TRANSPORT_ATP_SYNTHESIS_BY_CHEMIOSMOTIC_COUPLING_AND_HEAT_PRODUCTION_BY_UNCOUPLING_PROTEINS_ | -0.88 | 0 | Muscle |
| REACTOME_TCA_CYCLE_AND_RESPIRATORY_ELECTRON_TRANSPORT | -0.86 | 0 | Muscle |
| REACTOME_MUSCLE_CONTRACTION | -0.83 | 0 | Muscle |
| REACTOME_PYRUVATE_METABOLISM_AND_CITRIC_ACID_TCA_CYCLE | -0.83 | 0 | Muscle |
| KEGG_PARKINSONS_DISEASE | -0.81 | 0 | Muscle |
| KEGG_OXIDATIVE_PHOSPHORYLATION | -0.8 | 0 | Muscle |
| KEGG_CARDIAC_MUSCLE_CONTRACTION | -0.76 | 0 | Muscle |

## Table 3B. Gene Set Enrichment Analysis of the RNAseq data (GO, biological process)

| Gene ontology biological process | Enrichment score | FDR | Tissue enriched |
| --- | --- | --- | --- |
| CALCIUM_INDEPENDENT_CELL_CELL_ADHESION | 0.78 | 0 | Lung |
| DEFENSE_RESPONSE_TO_BACTERIUM | 0.71 | 0 | Lung |
| RESPONSE_TO_BACTERIUM | 0.7 | 0 | Lung |
| REGULATION_OF_T_CELL_ACTIVATION | 0.67 | 0 | Lung |
| LOCOMOTORY_BEHAVIOR | 0.66 | 0 | Lung |
| CELLULAR_DEFENSE_RESPONSE | 0.64 | 0 | Lung |
| T_CELL_ACTIVATION | 0.64 | 0 | Lung |
| RESPONSE_TO_VIRUS | 0.62 | 0 | Lung |
| RESPONSE_TO_OTHER_ORGANISM | 0.6 | 0 | Lung |
| CELL_ACTIVATION | 0.6 | 0 | Lung |
| LEUKOCYTE_ACTIVATION | 0.6 | 0 | Lung |
| DEFENSE_RESPONSE | 0.58 | 0 | Lung |
| INFLAMMATORY_RESPONSE | 0.58 | 0 | Lung |
| LYMPHOCYTE_ACTIVATION | 0.58 | 0 | Lung |
|  |  |  |  |
| CELLULAR_RESPIRATION | -0.88 | 0 | Muscle |
| AEROBIC_RESPIRATION | -0.88 | 0 | Muscle |
| ENERGY_DERIVATION_BY_OXIDATION_OF_ORGANIC_COMPOUNDS | -0.83 | 0 | Muscle |
| REGULATION_OF_MUSCLE_CONTRACTION | -0.79 | 0 | Muscle |
| FATTY_ACID_OXIDATION | -0.75 | 0.003 | Muscle |
| ENERGY_RESERVE_METABOLIC_PROCESS | -0.73 | 0.023 | Muscle |
| GENERATION_OF_PRECURSOR_METABOLITES_AND_ENERGY | -0.66 | 0 | Muscle |
| SKELETAL_MUSCLE_DEVELOPMENT | -0.66 | 0.006 | Muscle |
| MITOCHONDRIAL_TRANSPORT | -0.66 | 0.03 | Muscle |
| REGULATION_OF_HEART_CONTRACTION | -0.64 | 0.029 | Muscle |
| MUSCLE_DEVELOPMENT | -0.61 | 0 | Muscle |
| STRIATED_MUSCLE_DEVELOPMENT | -0.6 | 0.019 | Muscle |
| GLUCOSE_METABOLIC_PROCESS | -0.59 | 0.06 | Muscle |
| MITOCHONDRION_ORGANIZATION_AND_BIOGENESIS | -0.57 | 0.027 | Muscle |
| ELECTRON_TRANSPORT_GO_0006118 | -0.56 | 0.027 | Muscle |
| CELLULAR_PROTEIN_CATABOLIC_PROCESS | -0.52 | 0.053 | Muscle |
| PROTEIN_CATABOLIC_PROCESS | -0.5 | 0.054 | Muscle |
| CELLULAR_CARBOHYDRATE_METABOLIC_PROCESS | -0.47 | 0.05 | Muscle |
| CELLULAR_CATABOLIC_PROCESS | -0.45 | 0.05 | Muscle |
| CATABOLIC_PROCESS | -0.45 | 0.051 | Muscle |


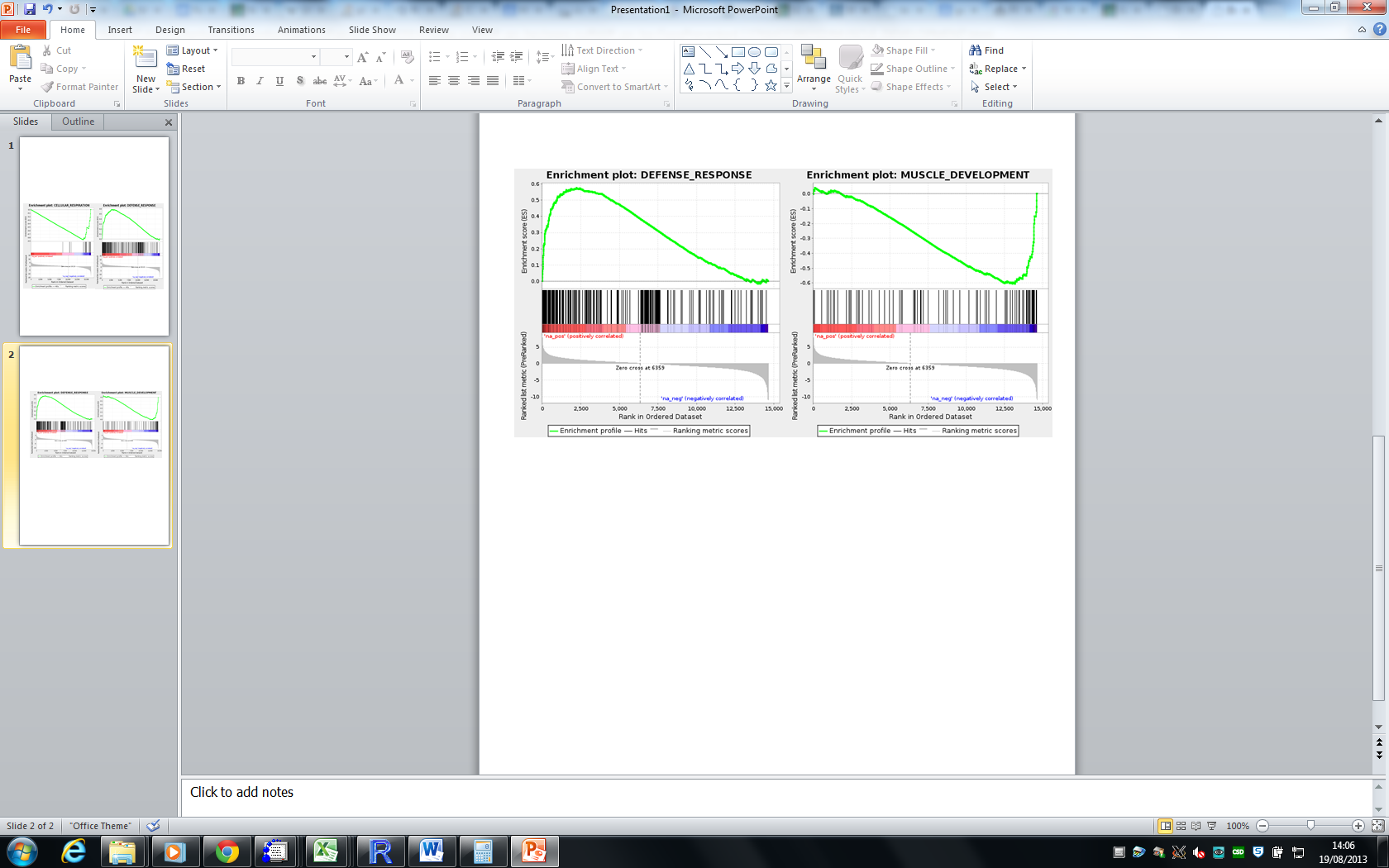


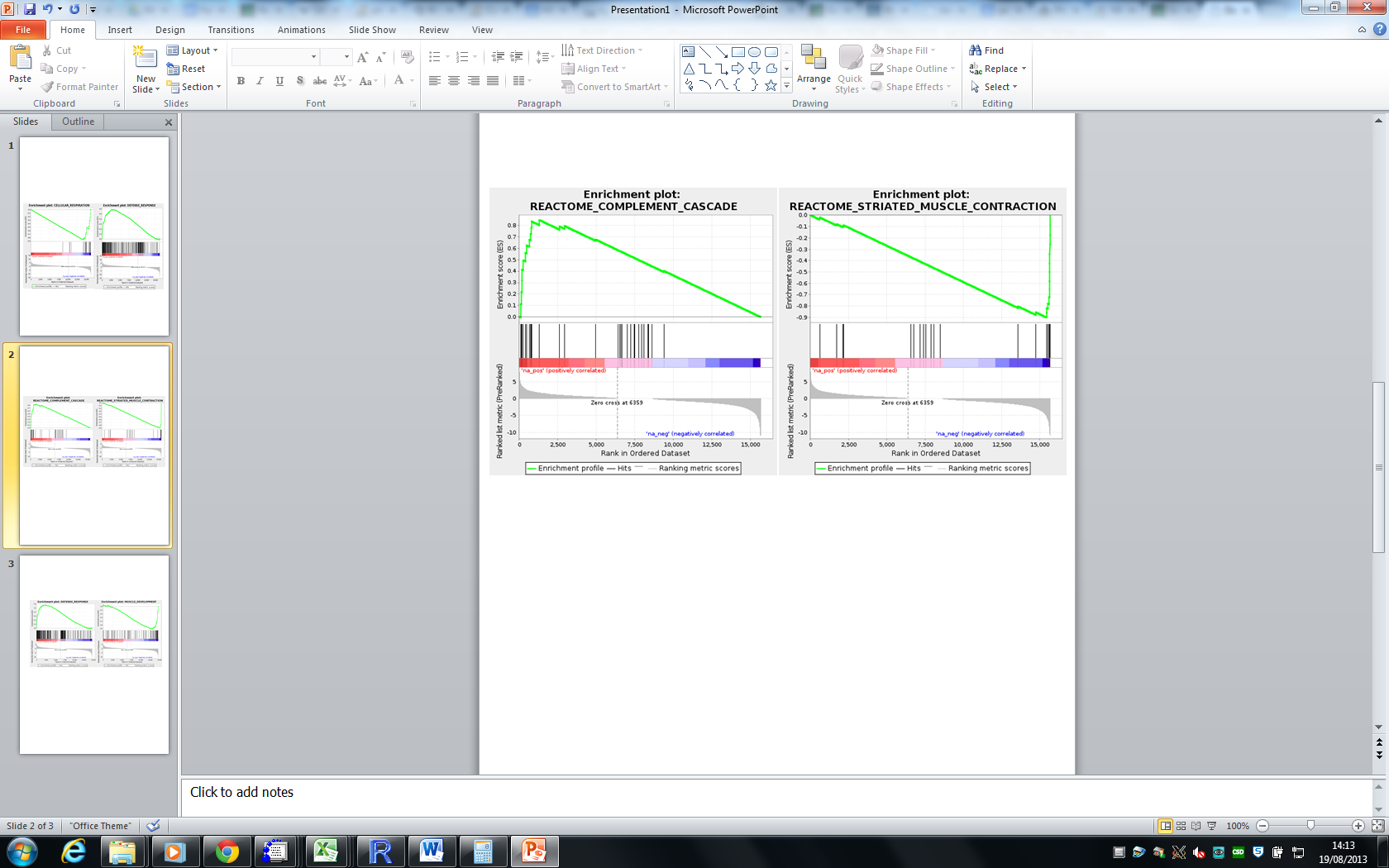


Figure S4. Pictorial representations of the GSEA.

## 1.6. Grouping of transcripts and evidence of novel transcript/gene discovery

An investigation into transcript redundancy, potential isoforms and if the RNAseq data provides new data was carried out. There is evidence that the February 2008 *Cavia porcellus* draft assembly (Broad Institute cavPor3) contains duplicated regions of chromosomes on different scaffolds. This is shown by the fact that some transcripts map to multiple scaffolds with exactly the same high quality alignment statistics. This seems biologically unlikely, as some polymorphism differences would be expected between genuine paralogs. One example is the transcript *lung_muscle_trans_00000002* that maps to 13 different scaffolds with 100% identity, over the full length of the 943 base pair sequence. This sequence does not span a known repeat. This provides good evidence of redundancy within the genome assembled transcriptome. The full assembly contained 151,072 transcripts and a subset of 86,044 was used in the design of 60,984 probes for the final custom 60K Agilent microarray. The full transcriptome sequences (**Davidsen_et_al_full_transcriptome.txt**) are accessible in the online material: http://pcwww.liv.ac.uk/~herberjm/Davidson_et_al/Additional_file6.zip.

To find potential isoforms of the same gene and to reduce redundancy within the transcriptome, two approaches were taken;

(1) Uclust from the Usearch algorithm was used to cluster sequences in a *de-novo* process [63]. This resulted in a set of 93,902 non-redundant transcripts, which represent the centroid sequence of clusters and any singletons. This was performed using a 90% identity threshold. A set of non-redundant sequences can be found in the online file **Daviden_et_al_uclust_sequences.txt** and the online file **Davidsen_et_al_Uclust_Bedtools_domains_genes.xlsx** contains sequence identifiers of clusters^[[2]](#footnote-2)^.

(2) Potential isoform groups were attained by genome position, by utilising the BEDTools [64] cluster algorithm that assigns overlapping transcripts in the genome to locus clusters. The resulting clusters and the transcripts they contain can also be found in the online supporting material as **Davidsen_et_al_Uclust_Bedtools_domains_genes.xlsx**.

From the BEDTools loci, there is evidence that the RNAseq data has identified novel genes or transcripts. From the 33,797 total distinct loci, the numbers of potential novel loci are as follows:

- Total no. of BEDTools cluster loci = 33,797
- No. of loci that contain an Ensembl cDNA = 16,392
- Number of loci that contained either an Ensembl cDNA or a Genscan prediction = 27,374
- Number of loci that contained purely public EST and/or mRNA data = 686
- Number of potential novel loci originating from the RNAseq data (if it contained at least one RNAseq data transcript but no Ensembl cDNAs) = 7,685
- Number of potential novel loci originating from the RNAseq data (that is, it contained at least one RNAseq transcript and no Ensembl cDNAs or Genscan gene predictions) = 5,737

This provides evidence that the RNA second generation sequencing data have produced a more comprehensive GP transcriptome when compared to Ensembl cDNAs and Genscan predictions. This has enabled a more robust investigation into the effects of smoking using this model. A set of 5,737 novel transcript loci were found and are labelled as ‘RNAseq_Novel’ in the BEDTools file (**Davidsen_et_al_Uclust_Bedtools_domains_genes.xlsx**) available online.

## 1.7 Novel functional domains added to the transcriptome

Further evidence for RNAseq novelty was found using a comparison of functional domain content between the RNAseq transcriptome data and Ensembl cDNA sequences. This was performed using the Hmmer package [65] with the Pfam protein families database A [66]. Initially, open reading frames from all six translation frames of each sequence was derived using the Sixpack tool of EMBOSS, lifted from the Iprscan suite [67,68]. An expectation value of 1e-6 threshold was employed running hmmsearch. There were 26,129 Ensembl cDNA transcripts in the cavPor3.64 genome, which consisted of 5,397 non-redundant Pfam A functional domains. In contrast, the number of transcripts built from the RNAseq data alone was 82,945 and these transcripts contained 5,478 distinct functional domains. This showed 81 potentially new functional domains as compared to the public data. However, the RNAseq data were derived from two distinct tissues of lung and muscle and some transcripts within the Ensembl cDNA data could be preferentially expressed in other tissues. This was evident when intersect and specific domain frequencies were taken. The number of domains specific to Ensembl cDNAs was 38 and 119 were specific to RNAseq data, with an intersect count of 5,359. A union total number of domains were 5,516 and all domains and classes can be viewed in the Domains sheet in **Davidsen_et_al_Uclust_Bedtools_domains_genes.xlsx** available online^[[3]](#footnote-3)^.

## 1.8 New representations of mouse orthologs from RNAseq transcripts

From the full transcriptome, prior to custom array design, transcripts were assigned mouse orthologs using blastx. 19,867 of 26,129 Ensembl cDNAs were assigned a mouse ortholog and this amounted to 16,537 distinct mouse genes. In comparison, from the 82,945 RNAseq derived transcripts, 52,513 were assigned a mouse ortholog and the number of non-redundant mouse genes was 17,347. The union, intersect and specific frequencies were as follows:

- Total mouse orthologs (union) of Ensembl cDNA and RNAseq data = 17,441
- Ensembl cDNA specific mouse orthologs = 94
- RNAseq specific mouse orthologs = 904
- Intersection between Ensembl cDNA and RNAseq mouse orthologs = 16,443

From this analysis, 904 potential new GP genes were found due to RNAseq sequencing. The actual gene symbols for each of the classes can be seen in the ‘MouseOrthologs_RNAseq_v_Ensembl’ sheet in the Excel file **Davidsen_et_al_Uclust_Bedtools_domains_genes.xlsx** available in the online supporting material.

**2.0 Development of a 60K Custom Microarray Design**

A total of 116,623 60-mer oligonucleotide probes were designed from the 151,072 transcript sequences summarised in Table 2 using the Agilent eArray software. These were used to design an initial 180K custom Agilent microarray in order to test the performance of this large set of capture probes. Pooled mRNA from GP lung and muscle samples (see Method section) were hybridised to these arrays.

The selection of probes for the final 60K array design was based on an assessment of the reliability of each probe compared to the sequence read counts in both tissues of interest. This was performed using a two-step process. For each tissue, sequencing read counts (reads per kilobase of transcripts per million (RPKM) transformed) and microarray probe intensities were LOESS normalised to generate a ratio representing the relative difference in signal intensity between both platform technologies. The ratios for each probe in lung and muscle were then compared by fitting a linear model (lm, R software), and the residual for each probe was calculated. The residuals represent an overall measure of the probe reliability when compared in both tissues, regardless of signal intensity. This approach assumes that the relationship between sequence counts and probe intensity is not proportional. Probes with similar differences between signal intensity and read counts in lung and muscle, even if this difference is relatively large, were still able to detect relative changes in mRNA concentrations between experimental groups. For each gene symbol mapped to the sequencing data, the selection of a probe to include on the final array design was done by selecting the probe with the lowest residual. The resulting correlation between technologies was mapped as a scatterplot for each of the two tissues, respectively (**Figures S5 and S6)**. The Pearson correlation and associated *P* value are indicated in the graphs. To avoid taking the log base 2 of zero, we added 1 to each of the counts prior to taking the log.


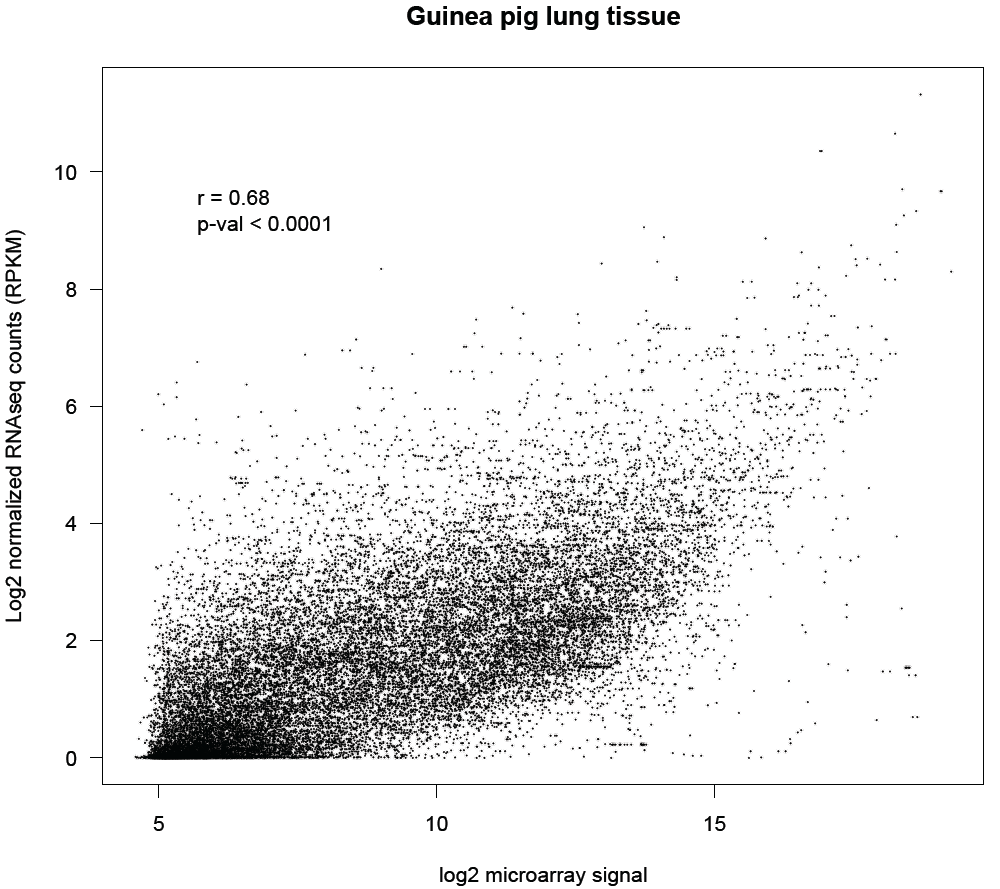


Figure S5.


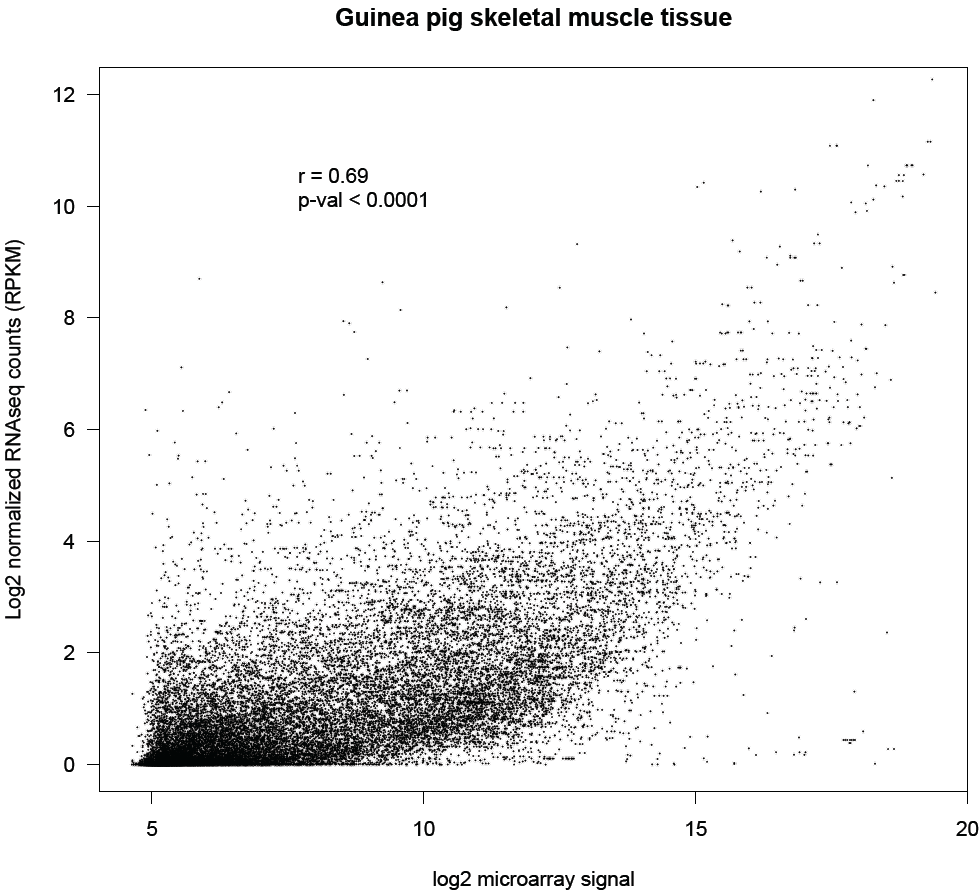


Figure S6.

We further illustrated the quantitative relationship of log_2_ fold-changes for each gene (lung *versus* muscle gene expression comparison) between RNAseq and our custom 60K microarray platform in the form of a scatterplot as shown in Figure S7.


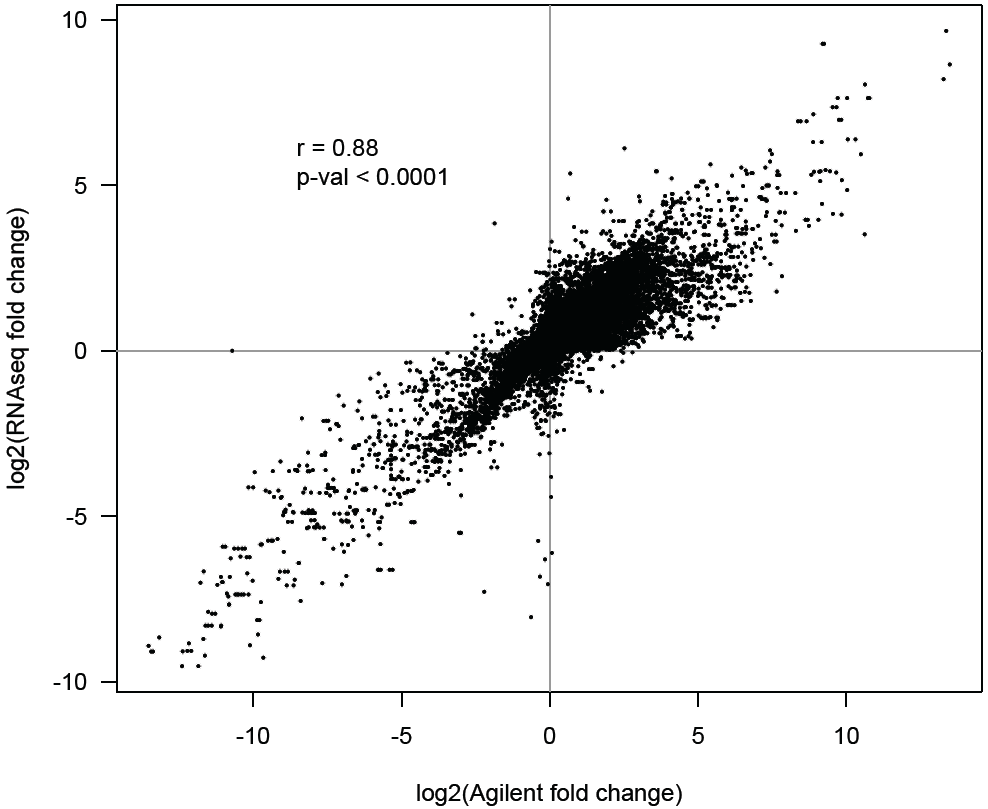


Figure S7. Comparison of estimated log2 fold-changes (lung/muscle) from Illumina RNAseq (y-axis) and the custom microarray platform (x-axis).

Finally, we also demonstrate that the lung specific genes detected using RNAseq technology (using multiple RPKM detection thresholds) are also detected specifically by our custom microarray platform on pooled cRNA from lung but not on pooled cRNA from muscle tissue (and vice versa) (see Figure S8 and S9).

Figure S8. Plot indicating the fractional overlap (y-axis) between profiling technologies in terms of genes being called lung-specific based on the RNAseq data (x-axis). The numeric value above each triangle indicates the number of transcripts termed lung-specific by RNAseq (at a given RPKM threshold) that overlap with genes being expressed above detection background in lung (blue triangles) and muscle tissue (red triangles), respectively, on our 60K custom microarray platform.

Figure S9. Plot indicating the fractional overlap (y-axis) between profiling technologies in terms of genes being called muscle-specific based on the RNAseq data (x-axis). The numeric value above each triangle indicates the number of transcripts termed lung-specific by RNAseq (at a given RPKM threshold) that overlap with genes being expressed above detection background in muscle (red triangles) and lung tissue (blue triangles), respectively, on our 60K custom microarray platform.

1. Supporting material is available online at http://pcwww.liv.ac.uk/~herberjm/Davidson_et_al/Additional_file6.zip [↑](#footnote-ref-1)
2. Supporting material is available online at http://pcwww.liv.ac.uk/~herberjm/Davidson_et_al/Additional_file6.zip [↑](#footnote-ref-2)
3. Supporting material is available online at http://pcwww.liv.ac.uk/~herberjm/Davidson_et_al/Additional_file6.zip [↑](#footnote-ref-3)
