# Supplementary material for: A systems biology approach reveals a link between systemic cytokines and skeletal muscle energy metabolism in a rodent smoking model and human COPD
Source: Genome Med. 2014 Aug 9;6(8):59. doi: 10.1186/s13073-014-0059-5 (PMC4165371; doi:10.1186/s13073-014-0059-5)
Supplement: Additional file 2 — Table with physiological characteristics of the clinical COPD cohort. Data are presented as means ± SEM. [file 13073_2014_59_MOESM2_ESM.docx]

Table with anthropometric characteristics defining the COPD cohort used in the paper:

|  | **Healthy controls** | **COPD, BMI_norm._** | **COPD, BMI_low_** |
| --- | --- | --- | --- |
| Gender (M/F) | 10/2 | 9/0 | 6/0 |
| Age (years) | 65.3±2.9 | 69.4±1.5 | 69.2±4.6 |
| BMI (kg/m^2^) | 26.3±1.1 | 27.4±1.4 | 19.7±1.0^**,††^ |
| FFMI (kg/m^2^) | 21.0±0.8 | 21.5±0.7 | 16.7±0.9^**,††^ |
| VE (L/min) | 71.2±5.6 | 40.5±3.6^***^ | 33.0±3.8^***^ |
| FEV_1_ (L) | 3.46±0.2 | 1.41±0.09^***^ | 1.21±0.21^***^ |
| FEV_1_/FVC (%) | 75.9±2.4 | 44.0±2.7^***^ | 39.5±4.5^***^ |
| RV (% of pred.) | 103.9±5.2 | 145.0±13.3 | 160.0±28.6^*^ |
| VO_2max_ (l•min^-1^•kg^-1^) | 22.3±1.4 | 13.9±1.7^**^ | 14.4±1.5^**^ |
| Peak power (W) | 117±8 | 60±7^***^ | 47±9^***^ |
| 6MWD (m) | 584±24 | 469±30^*^ | 367±59^***^ |
| BODE index | 0.1±0.1 | 2.3±0.4^**^ | 4.0±1.0^***,†^ |
|  |  |  |  |
|  |  |  |  |
| Data are presented as mean±SEM.  **P* <0.05; ***P* <0.01; ****P* <0.001 *versus* controls. ^†^*P* <0.05; ^††^*P* <0.01; ^†††^*P* <0.001 *versus* COPD patients with a normal BMI. Comparisons were analysed using one-way ANOVA and Tukey’s *post hoc* test.  BMI: body mass index; FEV_1_: forced expiratory volume in 1 s; FFMI: fat-free mass index; VE: lung ventilation; 6MWD: 6-min walking distance | | | |
