# Supplementary material for: A systems biology approach reveals a link between systemic cytokines and skeletal muscle energy metabolism in a rodent smoking model and human COPD
Source: Genome Med. 2014 Aug 9;6(8):59. doi: 10.1186/s13073-014-0059-5 (PMC4165371; doi:10.1186/s13073-014-0059-5)
Supplement: Additional file 3 — Analysis of the genome-wide transcriptional response in lung tissue from COPD patients, healthy smokers and healthy non-smokers, respectively (GSE19407). [file 13073_2014_59_MOESM3_ESM.pptx]

## Slide 1
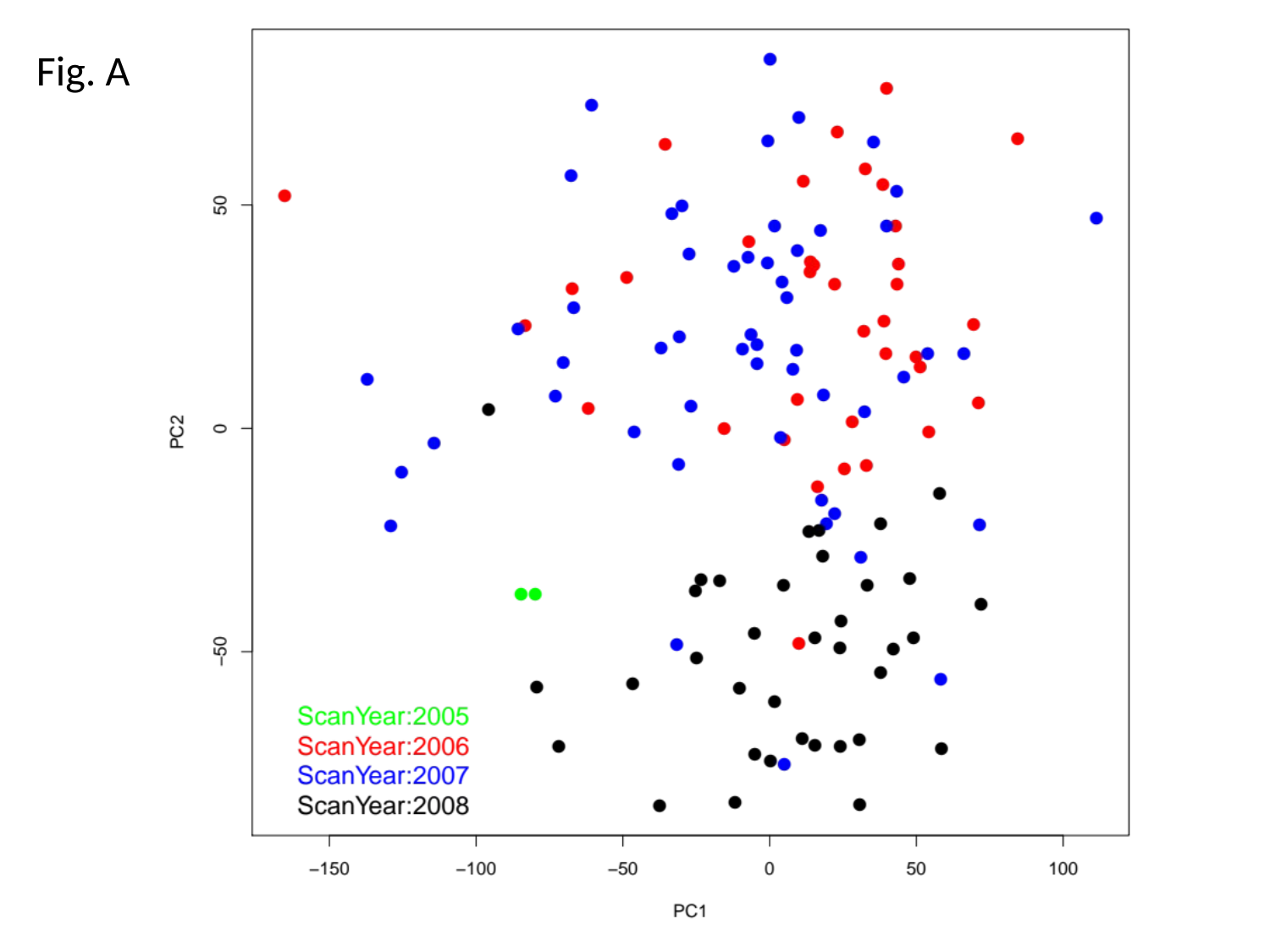

Fig. A

## Slide 2
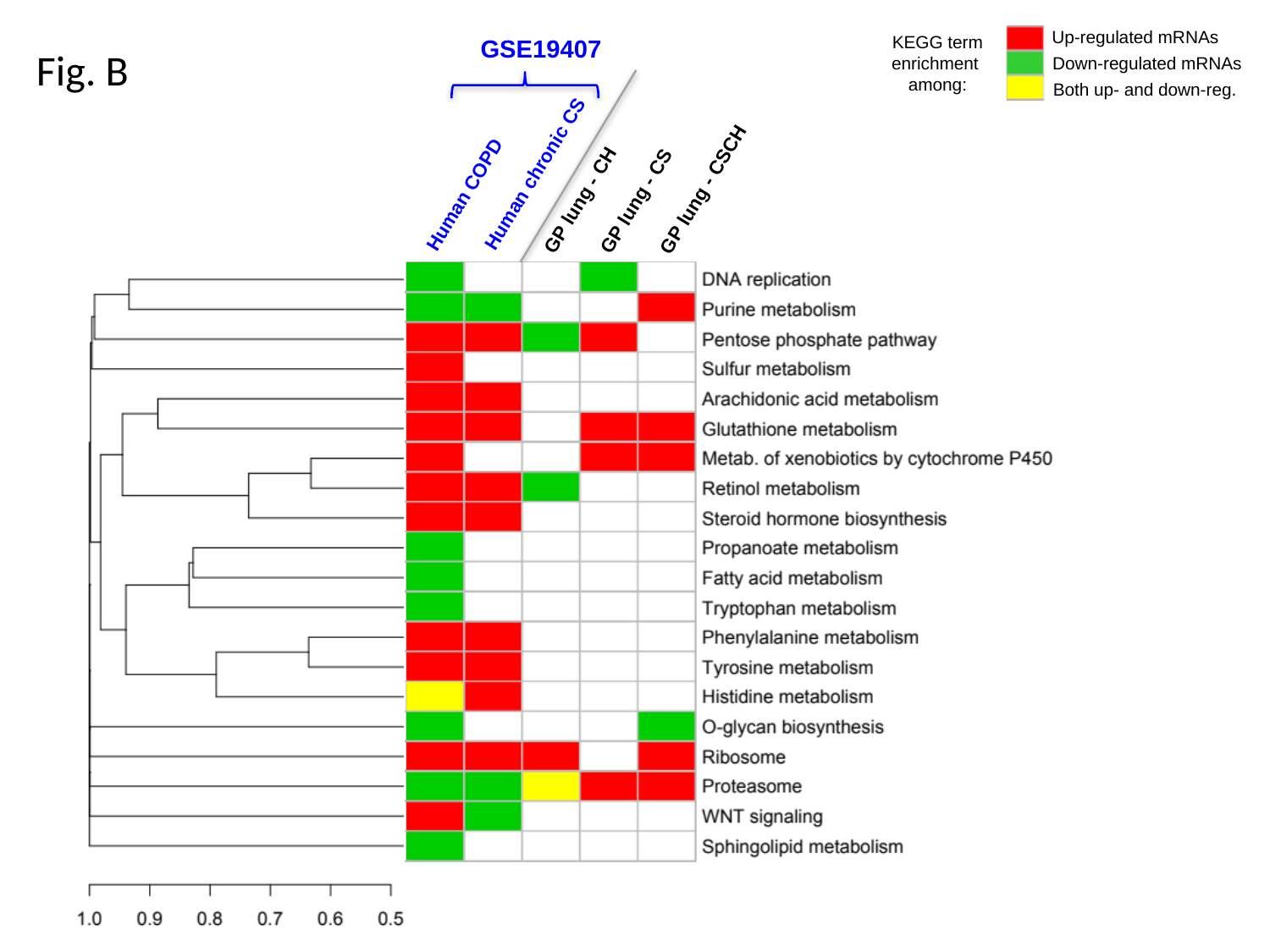

Up-regulated mRNAs
KEGG term
enrichment
among:
GSE19407
Human chronic CS
GP lung - CSCH
Human COPD
GP lung - CH
GP lung - CS
Fig. B
Down-regulated mRNAs
Both up- and down-reg.

## Slide 3
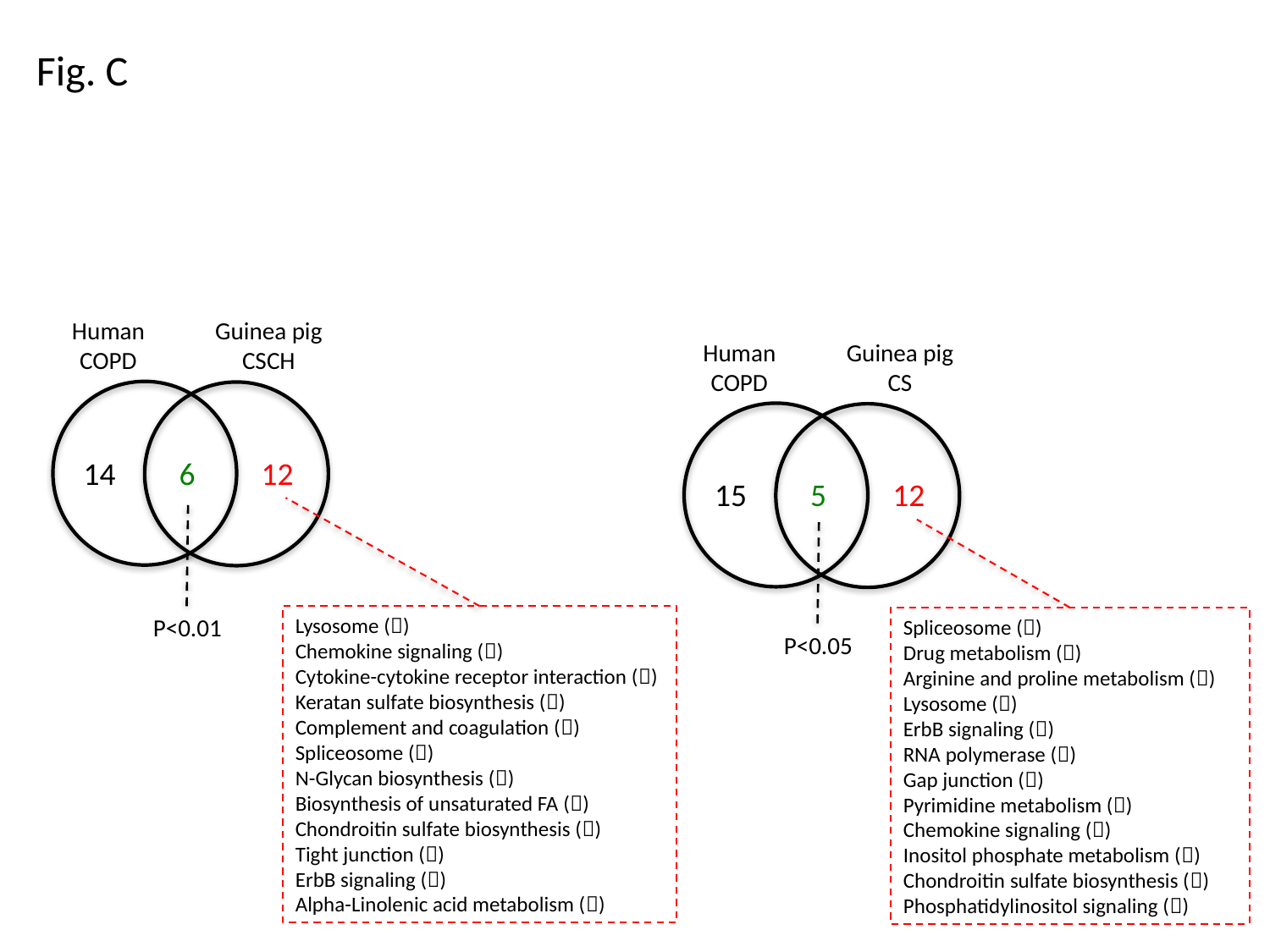

Fig. C
Human
COPD
Guinea pig
CSCH
14
6
12
Human
COPD
Guinea pig
CS
15
5
12
Lysosome ()
Chemokine signaling ()
Cytokine-cytokine receptor interaction ()
Keratan sulfate biosynthesis ()
Complement and coagulation ()
Spliceosome ()
N-Glycan biosynthesis ()
Biosynthesis of unsaturated FA ()
Chondroitin sulfate biosynthesis ()
Tight junction ()
ErbB signaling ()
Alpha-Linolenic acid metabolism ()
P<0.01
Spliceosome ()
Drug metabolism ()
Arginine and proline metabolism ()
Lysosome ()
ErbB signaling ()
RNA polymerase ()
Gap junction ()
Pyrimidine metabolism ()
Chemokine signaling ()
Inositol phosphate metabolism ()
Chondroitin sulfate biosynthesis ()
Phosphatidylinositol signaling ()
P<0.05
