# Supplementary figures and images for: A systems biology approach reveals a link between systemic cytokines and skeletal muscle energy metabolism in a rodent smoking model and human COPD
Source: Genome Med. 2014 Aug 9;6(8):59. doi: 10.1186/s13073-014-0059-5 (PMC4165371; doi:10.1186/s13073-014-0059-5)

## Slide 1
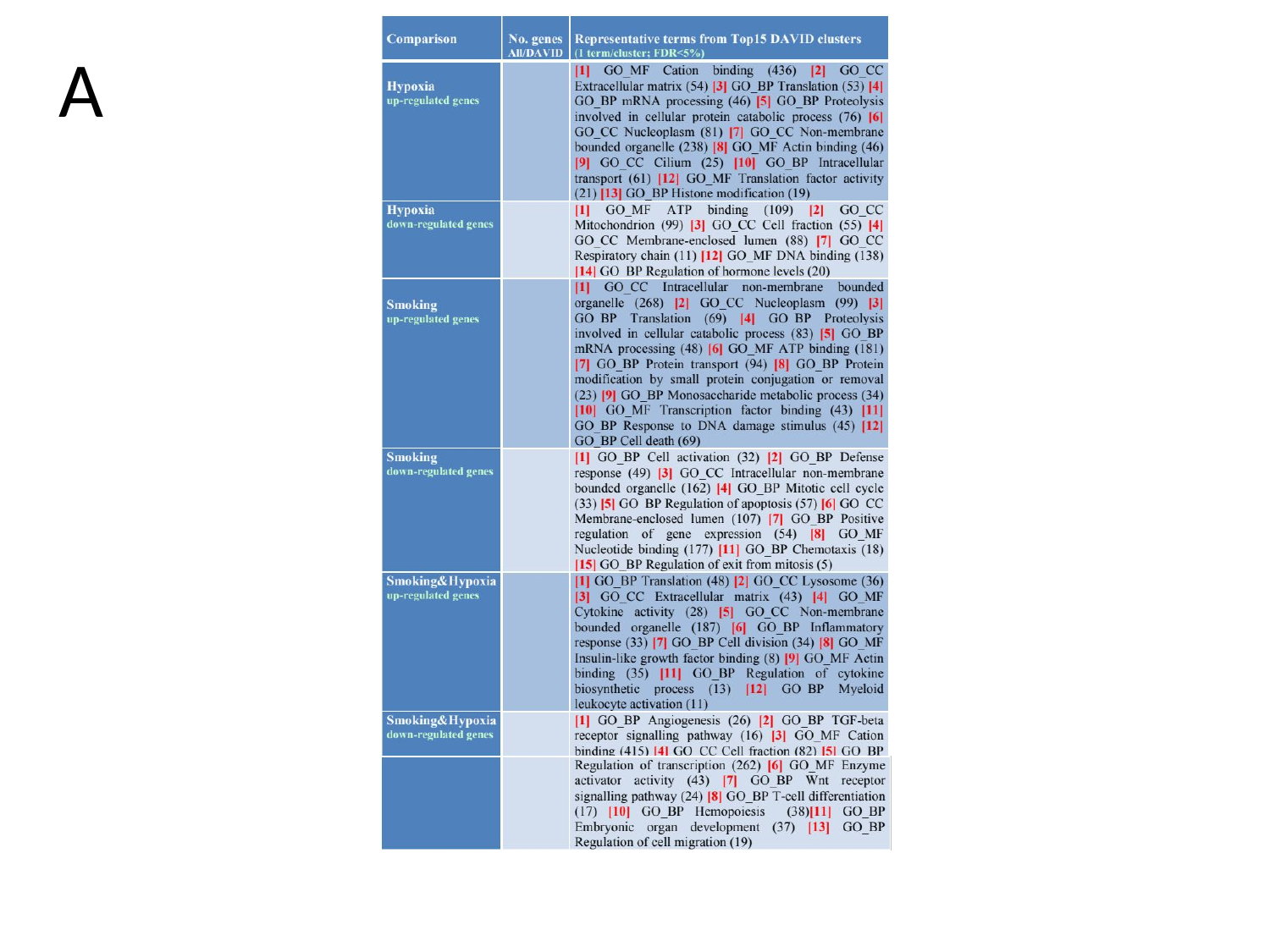

# A

## Slide 2
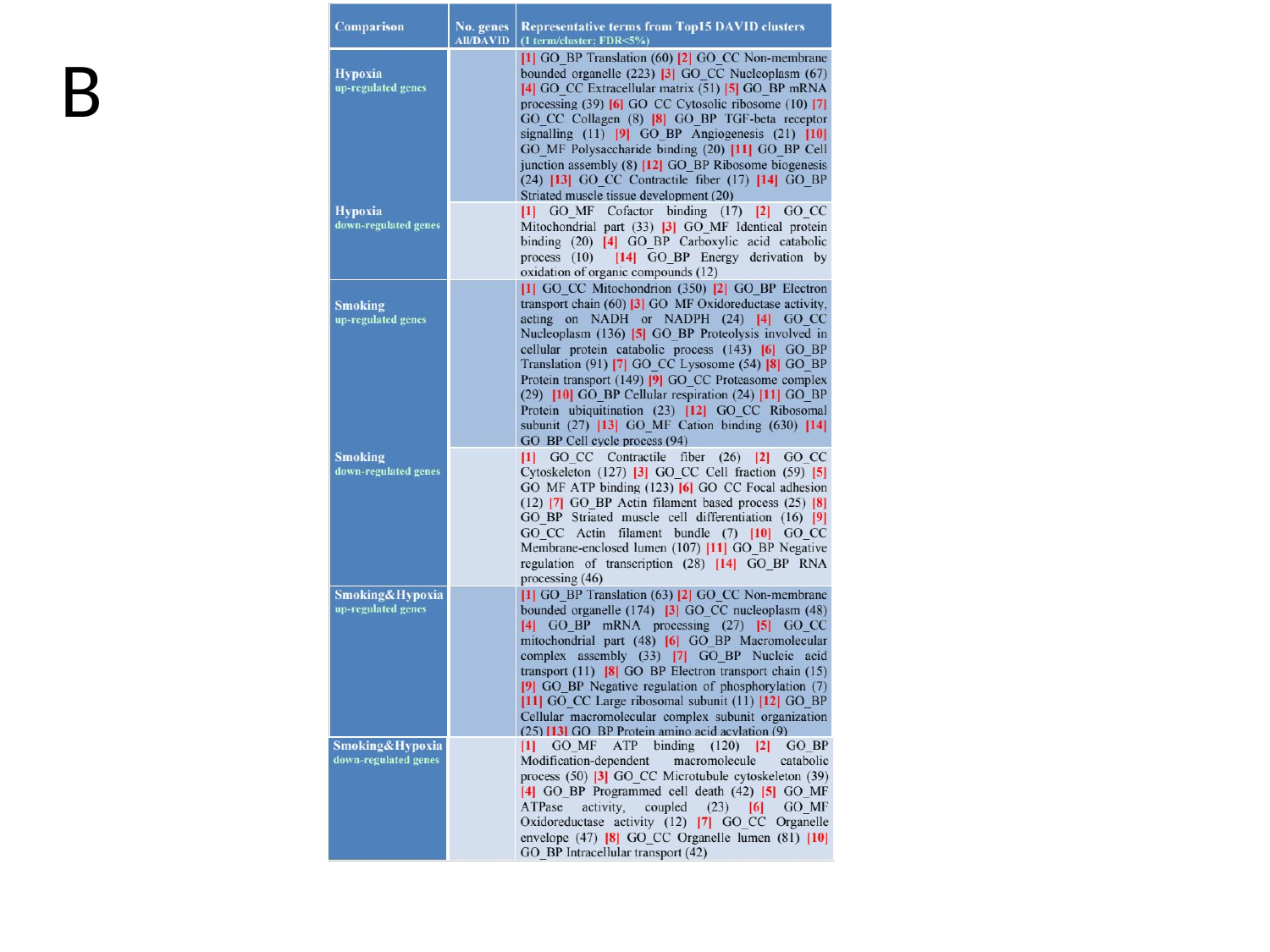

# B

## Slide 3
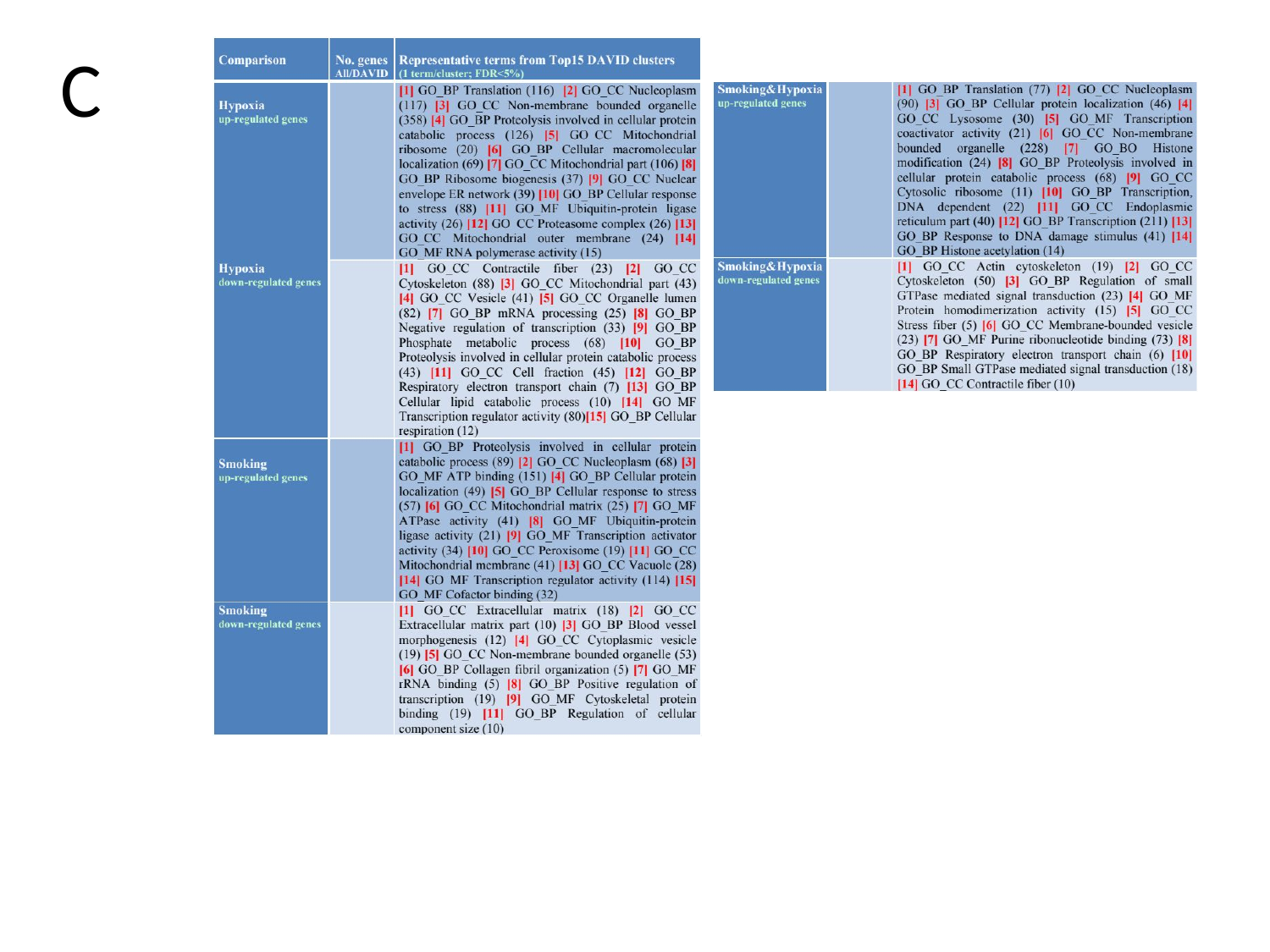

# C

Supplement: Additional file 7 — Lists of statistically enriched Gene Ontology terms (FDR <5%) in the different experimental conditions in guinea pig lung (A) and hindlimb muscles (soleus: B; gastro : C), respectively. Numbers in red squared brackets indicate the specific cluster number from the output of DAVID. The black open brackets give the number of enriched genes for a given ontology term. [file 13073_2014_59_MOESM7_ESM.pptx]

## Slide 1
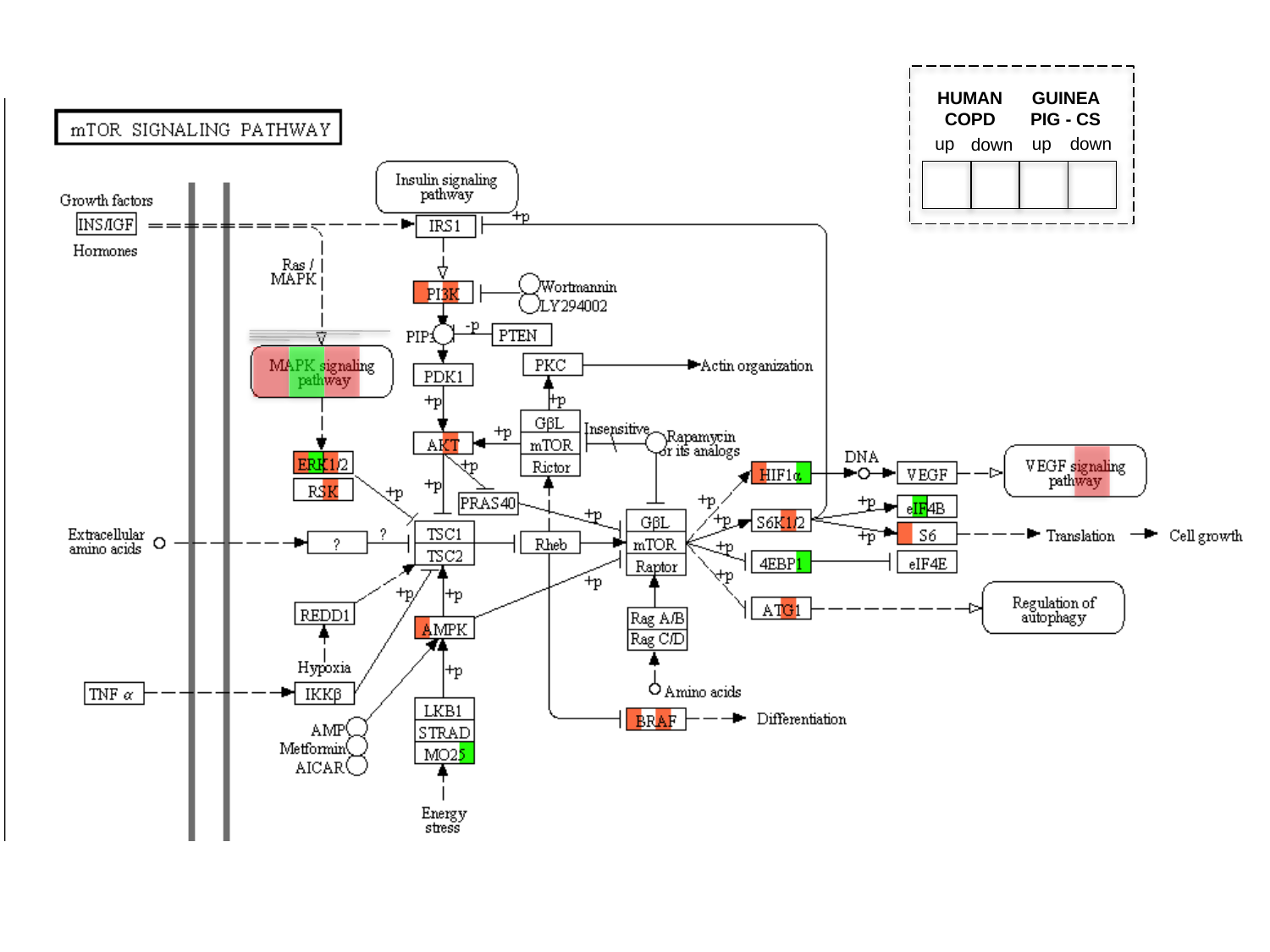

GUINEA PIG - CS
HUMAN
COPD
up
down
up
down

## Slide 2
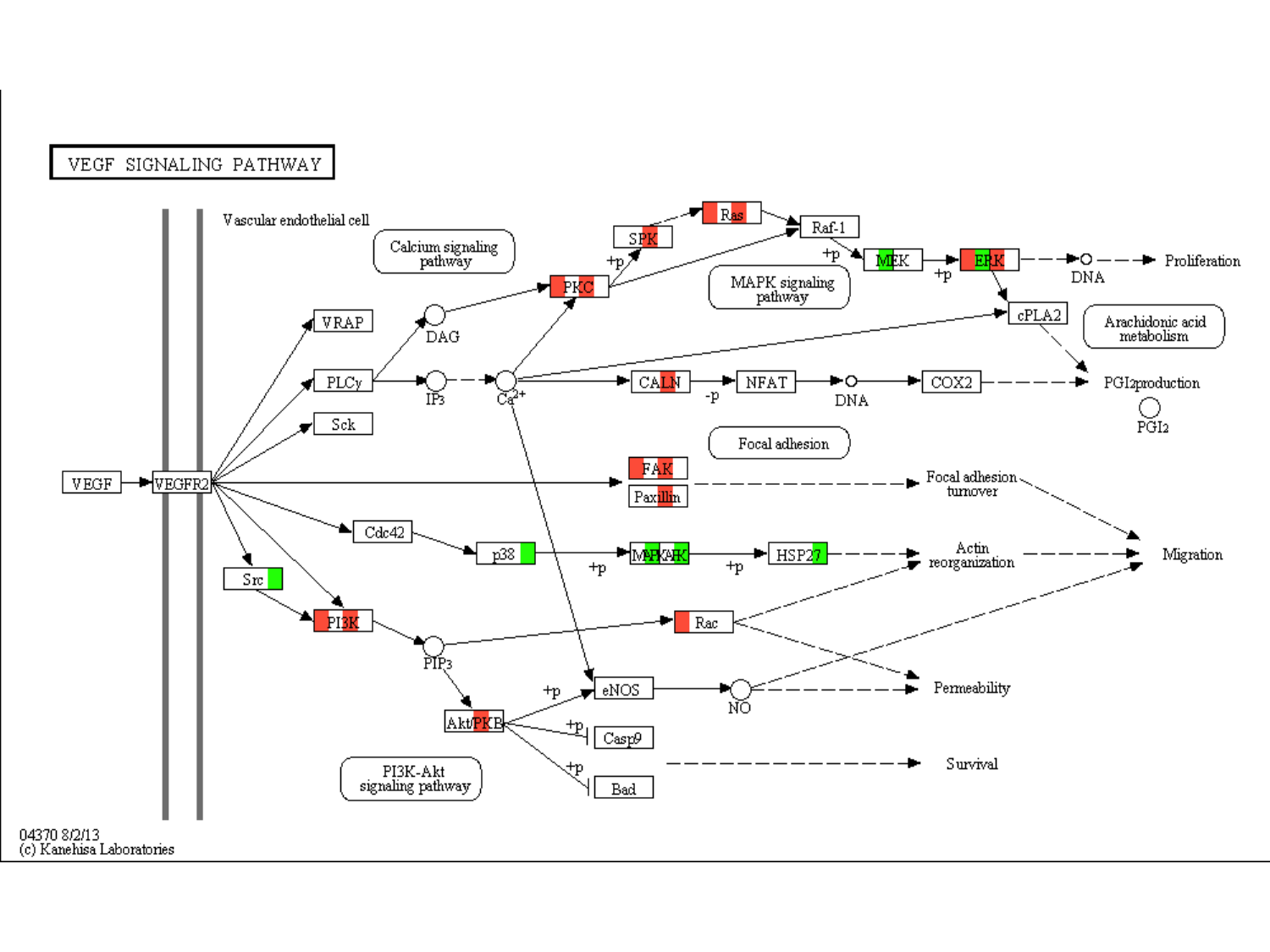

## Slide 3
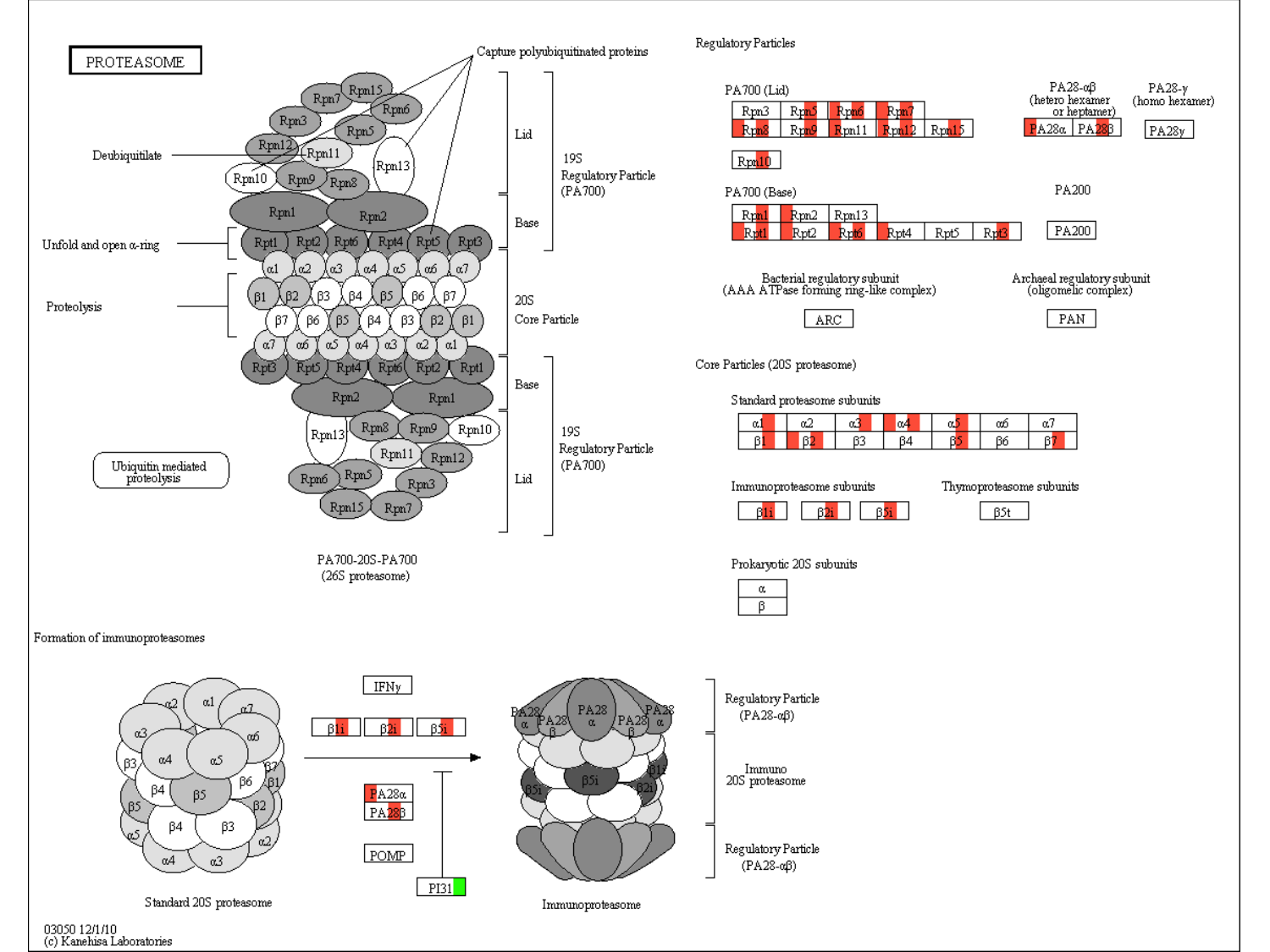

## Slide 4
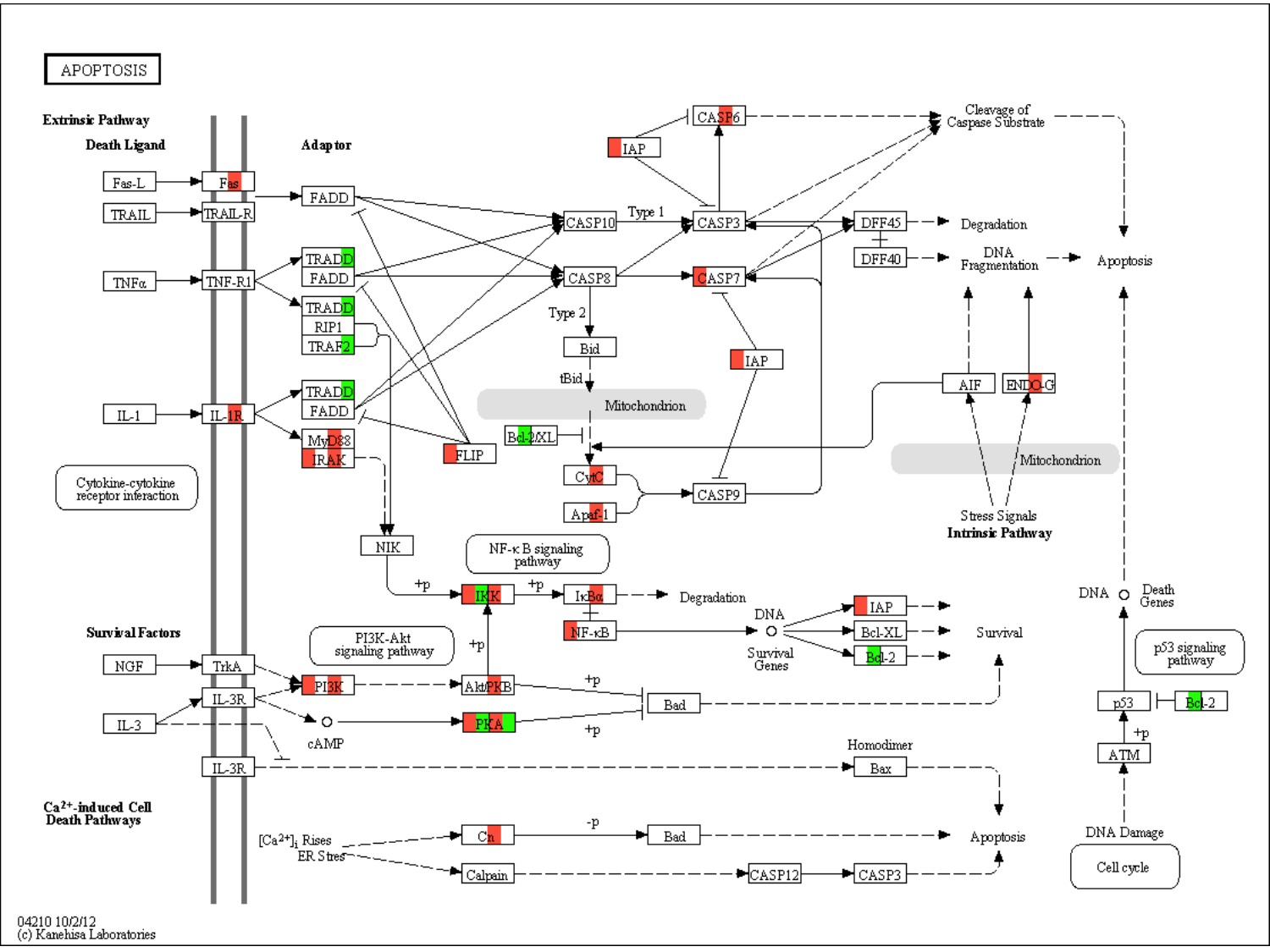

## Slide 5
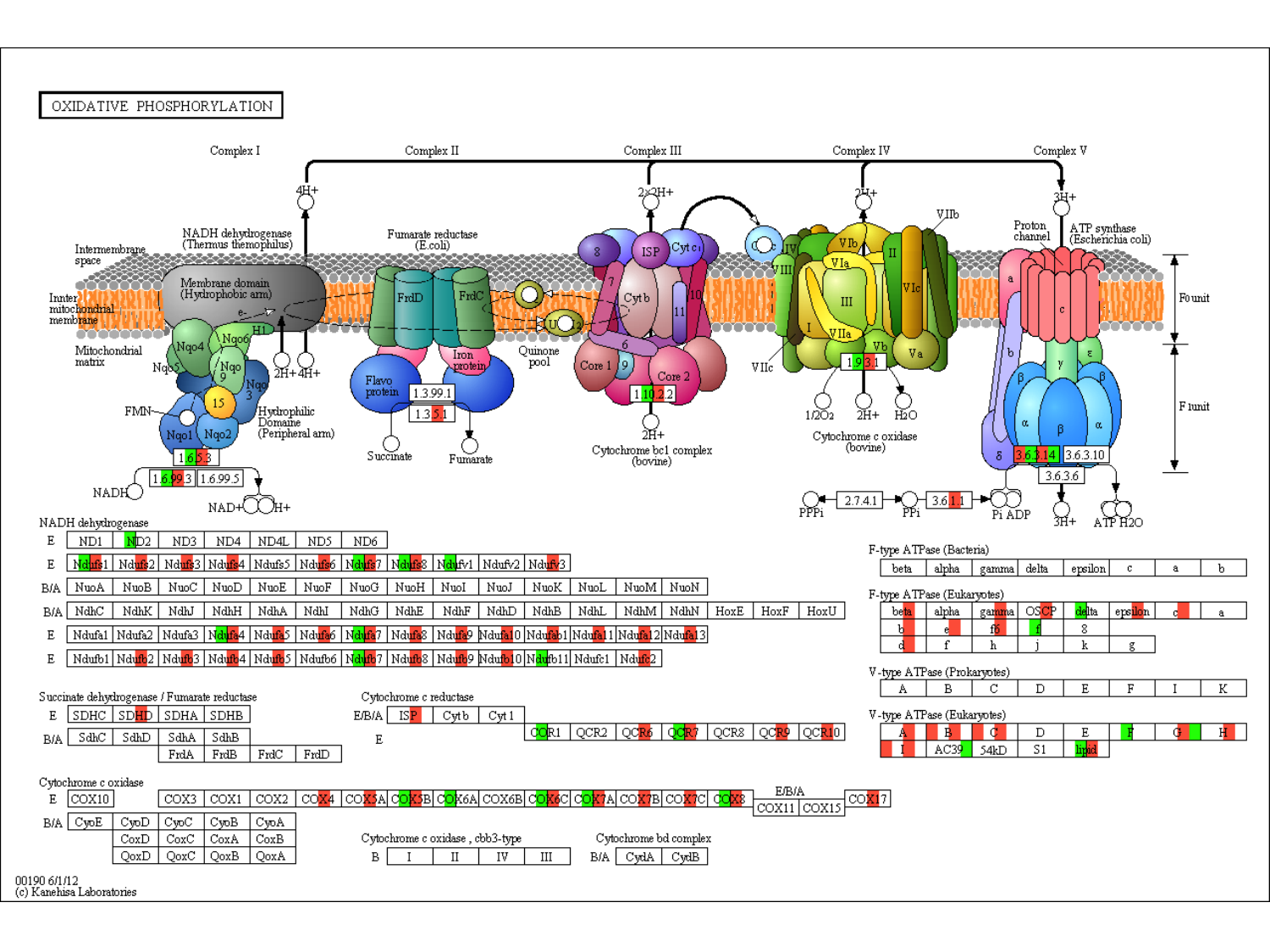

Supplement: Additional file 8 — KEGG pathway diagrams for five key pathways that are functionally enriched in soleus muscle of guinea pigs exposed to long-term CS. Each gene in a pathway has been divided into four sectors in order to demonstrate how that gene is regulated in the guinea pig model as well as in the limb muscle of COPD patients when compared to their respective controls. Green indicates downregulation; red indicates upregulation. [file 13073_2014_59_MOESM8_ESM.pptx]
