# Supplementary material for: A systems biology approach reveals a link between systemic cytokines and skeletal muscle energy metabolism in a rodent smoking model and human COPD
Source: Genome Med. 2014 Aug 9;6(8):59. doi: 10.1186/s13073-014-0059-5 (PMC4165371; doi:10.1186/s13073-014-0059-5)
Supplement: Additional file 9 — CXCL9 (A) and CXCL10 (B) mRNA levels in lung tissue of sham controls (CON), cigarette smoke-exposed (CS), hypoxic (CH) and to combined stimuli (CSCH). Values (means ± SE (n = 3-4 guinea pig/group)) in the treated groups are presented as relative to the untreated sham controls (basal level = 1). * Significantly different from the control group (P <0.05). (*) tends to be significantly different among treated (0.05 > P <0.1). [file 13073_2014_59_MOESM9_ESM.docx]

*Validation of microarray results by real-time RT-PCR*

For validation of the novel custom microarray, we selected CXCL9 and CXCL10 as they were reported to be differentially regulated in exposed lung tissue by the array technology as well as being central in the gene regulatory network linking the lung and muscle compartments (Figure 5).

Consistent with the microarray results, CXCL10 is induced by CSCH and CH, respectively (Additional file 14). Further, the qPCR data confirm that CXCL9 mRNA expression is significantly repressed by CS and CSCH, respectively. The only experimental condition that could not be verified by qPCR was the reported induction of CXCL9 by CH (Additional file 14) due to intragroup variability in the qPCR data. Hence, we conclude that the qPCR results show good agreement with the custom microarray platform.
