# Supplementary material for: A systems biology approach reveals a link between systemic cytokines and skeletal muscle energy metabolism in a rodent smoking model and human COPD
Source: Genome Med. 2014 Aug 9;6(8):59. doi: 10.1186/s13073-014-0059-5 (PMC4165371; doi:10.1186/s13073-014-0059-5)
Supplement: Additional file 10 — Heatmap visualising the serum protein levels of selected cytokines in the human COPD cohort with the extrapulmonary focus (GSE27536). Each row represents a cytokine, whereas each column represents a human subject. Red colours mean increased expression whereas green colours mean decreased expression. An asterix denotes significance at P <0.05 for the disease factor (Mack-Skillings test). [file 13073_2014_59_MOESM10_ESM.pptx]

## Slide 1
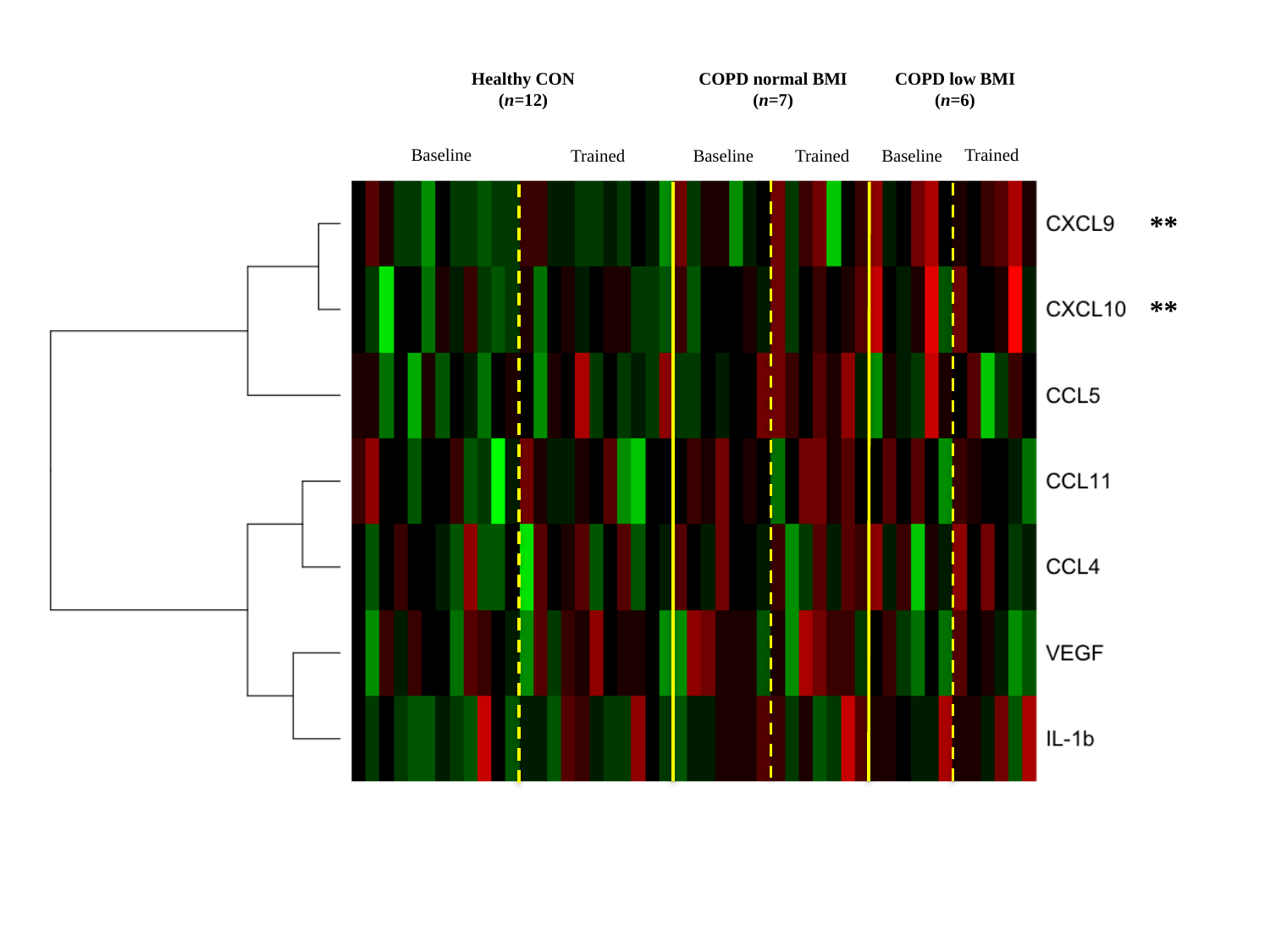

Healthy CON
(n=12)
COPD normal BMI
(n=7)
COPD low BMI
(n=6)
Baseline
Trained
Trained
Trained
Baseline
Baseline
**
**
