# Supplementary material for: A systems biology approach reveals a link between systemic cytokines and skeletal muscle energy metabolism in a rodent smoking model and human COPD
Source: Genome Med. 2014 Aug 9;6(8):59. doi: 10.1186/s13073-014-0059-5 (PMC4165371; doi:10.1186/s13073-014-0059-5)
Supplement: Additional file 11 — Scatterplots highlighting that the negative associations between CXCL9/-10 serum protein levels and muscle aerobic energy metabolism genes in the human COPD cohort are independent of exercise tolerance. Data have been standardised (z-scored). [file 13073_2014_59_MOESM11_ESM.docx]

Figure 1. VO_2_max differences between experimental groups in the clinical COPD cohort. The healthy age-matched controls had significantly higher VO_2_max levels than both disease groups (*P* <0.001) - irrespective of exercise training status. VO_2_max levels did not differ significantly between the two COPD groups. The green bars indicate the increase in VO2max following completion of an 8-week endurance exercise training programme. Error bars indicate SEM.


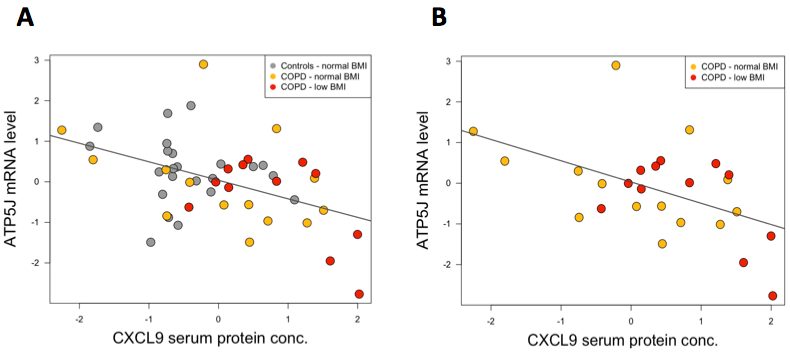


Figure 2. Correlation between z-scored standardised CXCL9 serum protein levels and the transcriptional abundance of ATP5J, a gene encoding for a protein in the fifth protein complex of oxidative phosphorylation. (A) All three patient groups were plotted (*R* = -0.46; *P* = 0.001). (B) When we removed the healthy control samples we still see a significant linear association (*P* = 0.01) between CXCL9 and ATP5J expression.


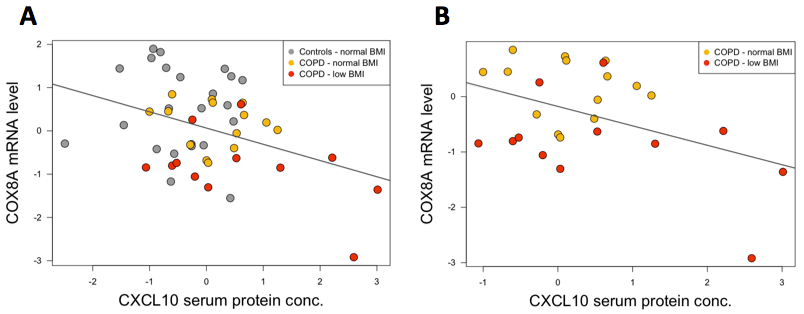


Figure 3. Correlation between z-scored standardised CXCL10 serum protein levels and the transcriptional abundance of COX8A, a gene encoding for a protein in the forth protein complex of oxidative phosphorylation. (A) All three patient groups were plotted (*R* = -0.37; *P* = 0.008). (B) When we removed the healthy control samples we still see a significant linear association (*P* = 0.03) between CXCL10 and COX8A expression.
