# Supplementary material for: A systems biology approach reveals a link between systemic cytokines and skeletal muscle energy metabolism in a rodent smoking model and human COPD
Source: Genome Med. 2014 Aug 9;6(8):59. doi: 10.1186/s13073-014-0059-5 (PMC4165371; doi:10.1186/s13073-014-0059-5)
Supplement: Additional file 12 — Scatterplots relating the distance walked in 6 min to the serum protein levels of CXCL9 and CXCL10, respectively. [file 13073_2014_59_MOESM12_ESM.pptx]

## Slide 1
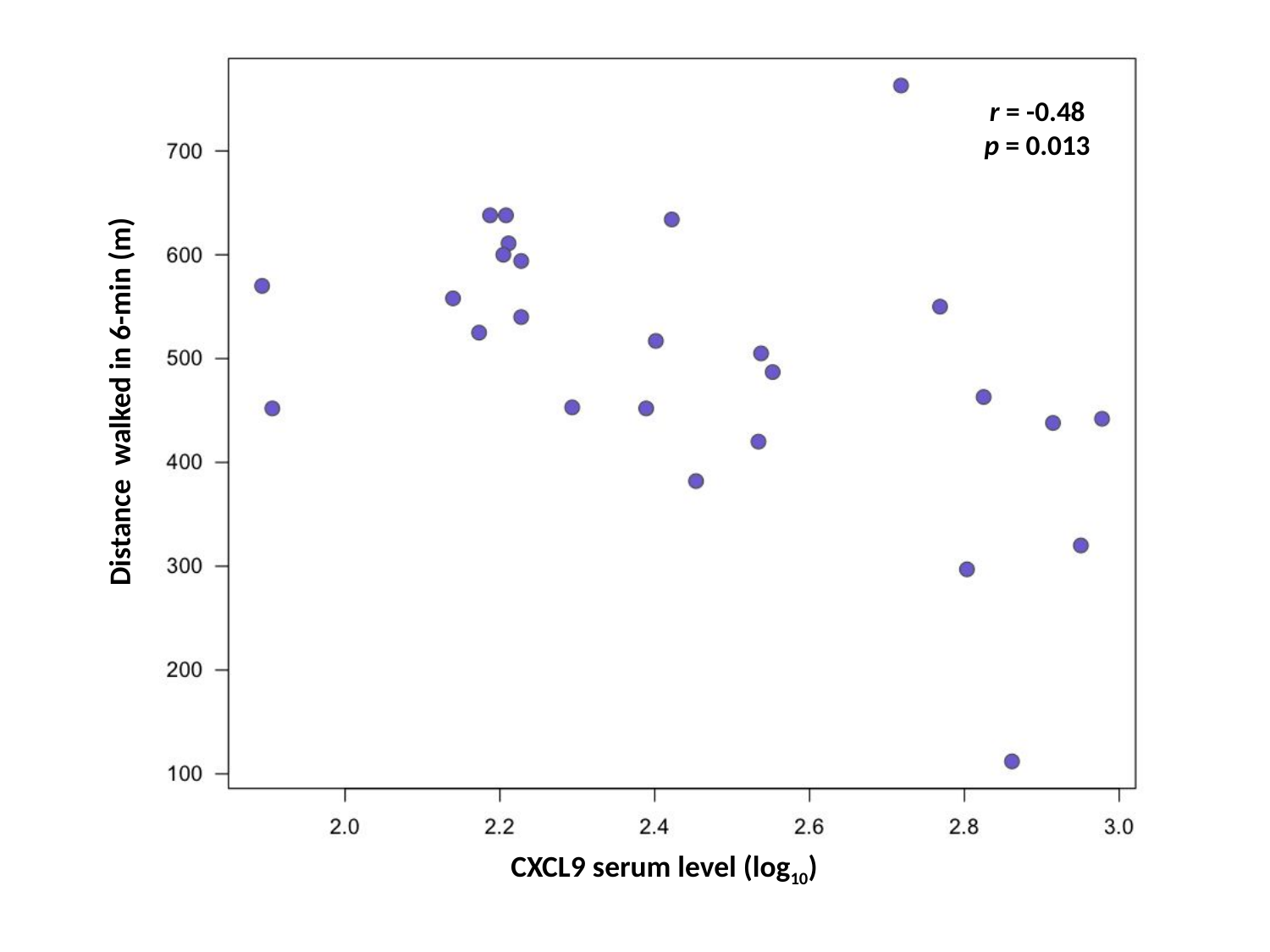

r = -0.48
p = 0.013
Distance walked in 6-min (m)
CXCL9 serum level (log10)

## Slide 2
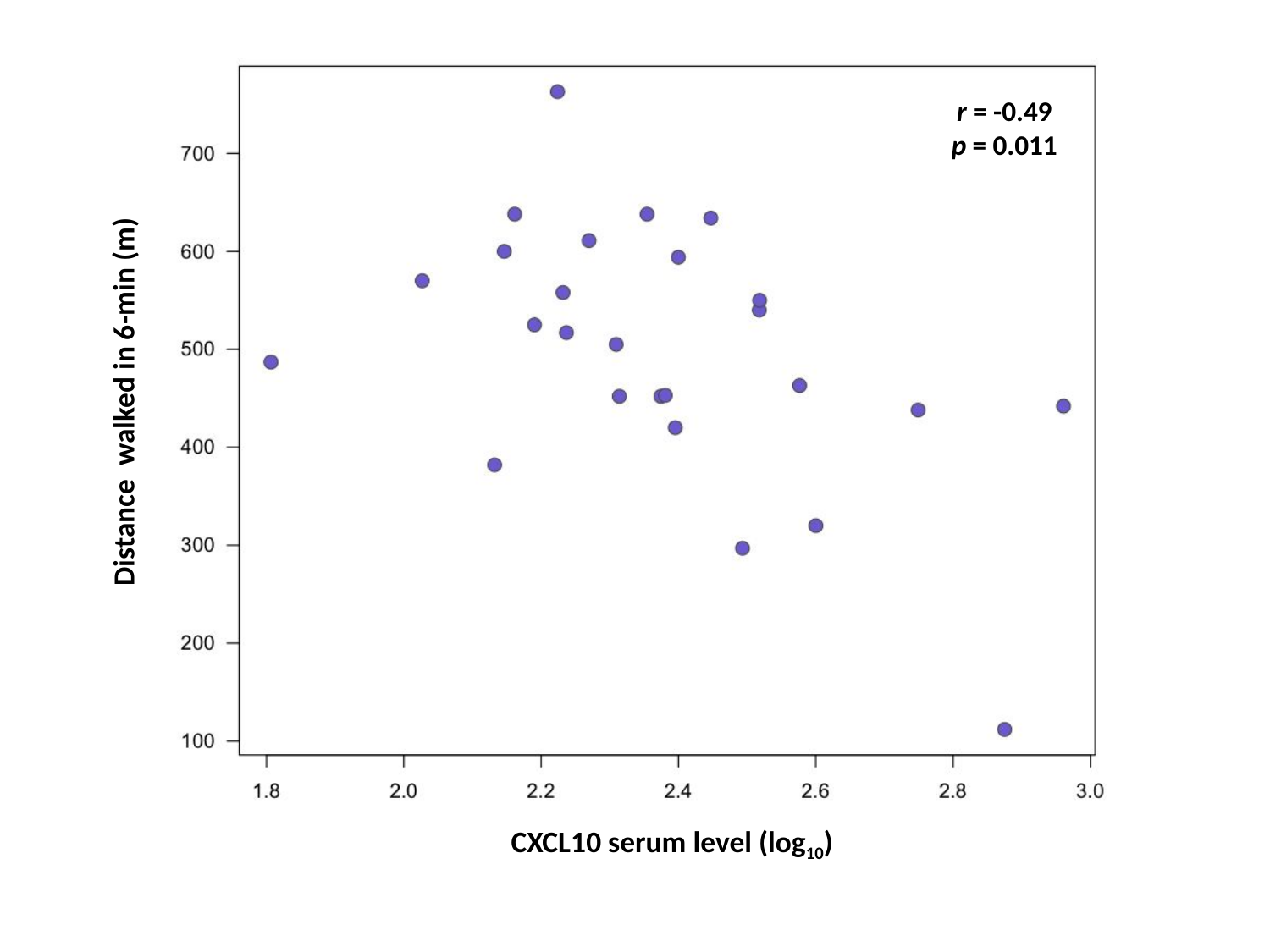

r = -0.49
p = 0.011
Distance walked in 6-min (m)
# CXCL10 serum level (log10)
