# Supplementary material for: A systems biology approach reveals a link between systemic cytokines and skeletal muscle energy metabolism in a rodent smoking model and human COPD
Source: Genome Med. 2014 Aug 9;6(8):59. doi: 10.1186/s13073-014-0059-5 (PMC4165371; doi:10.1186/s13073-014-0059-5)
Supplement: Additional file 13 — Analysis of the genome-wide transcriptional response of hindlimb skeletal muscle in C57 mice exposed to long-term nose-only cigarette smoke [ 24,70,71 ]. [file 13073_2014_59_MOESM13_ESM.docx]

**ANALYSIS OF SKELETAL MUSCLE TRANSCRIPTIONAL RESPONSE IN A MOUSE SMOKING MODEL**

**Summary**

In order to assess whether the extrapulmonary transcriptional response observed in guinea pigs exposed to smoking was conserved in the popular mouse smoking model, we analysed a publicly available microarray dataset representing the temporal transcriptional changes in the gastrocnemius muscle of nose-only CS or clean air (sham controls) exposed adult mice.

**Methods**

**Animal model**

In brief, 4-month-old female C57/BL6 mice received nose-only exposure of 4% mainstream CS or clean air for 2 h/day, 5 days a week for 2 (n = 6), 12 (n = 6) or 24 (n = 6) weeks, respectively. Twenty hours following the final smoke/air exposure, mice were anaesthetised with isoflurane, exsanguinated by cardiac puncture, and the gastrocnemius muscle was collected. For more information on RNA isolation as well as the labelling and hybridisation protocols, see GEO accession: GSE18033.

**Data processing and analysis**

Data processing of the raw Affymetrix CEL files was undertaken using Bioconductor in R. The arrayMvout package in R [70], which is based on dimension reduction of diverse established quality metrics, identified eight of the 38 arrays relevant for the cross-species comparison as technical outliers at a nominal outlier flagging rate of *α* = 0.05 (GSM450983, GSM450998, GSM451003, GSM451006, GSM451012, GSM451013, GSM451014, GSM451018). Hence, these eight samples were removed prior to RMA normalization (‘affy’ R package). Microarray data were then de-noised by removing probes for which 75% or more of the samples were flagged as ‘absent’ by the MAS5 algorithm (13,051 probes removed).

**Results**

**Transcriptional regulation in response to prolonged CS exposure can only be detected at a high FDR threshold**

In order to assess if exposure to long-term CS induced a significant transcriptional response, we set to identify differentially expressed genes. In order to identify genes linked to both time and experimental intervention (that is, CS *versus* sham controls) we used a two-factor ANOVA (Table 1). While we could identify thousands of genes changing across time, only 14 genes were differentially expressed between shams and CS-exposed mice at a standard FDR cutoff (FDR <15%) (Table 1). By increasing the cutoff to FDR <30% we could identify 303 modulated transcripts. These were subdivided in 129 and 174 genes up- and downregulated, respectively, based on hierarchical clustering (Figure 2). We then performed a functional enrichment analysis on these 303 genes using the web based tool DAVID [71].

| *Factor:* | **5% FDR** | **15% FDR** | **30% FDR** |
| --- | --- | --- | --- |
| Group | 5 | 14 | 303 |
| Time | 1,774 | 3,769 | 6,217 |
| Interaction | 0 | 14 | 375 |

Table 1. Number of differentially expressed genes at various statistically thresholds identified via a two-way ANOVA in R.


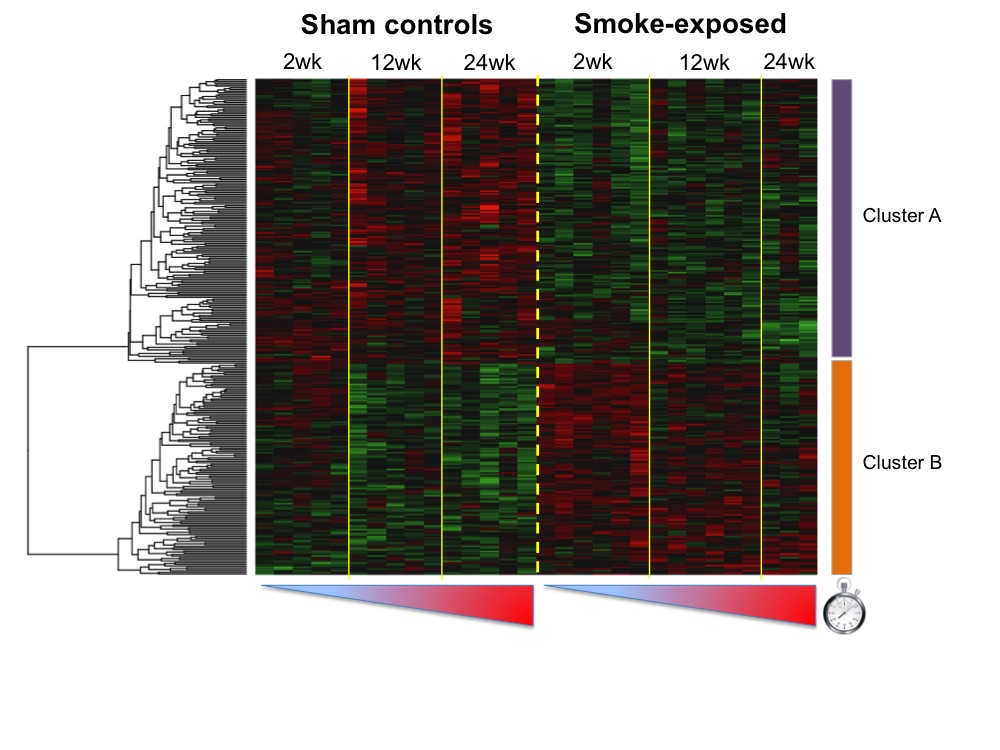


Figure 2. Heatmap of the 303 genes that are differentially expressed between shams and smoke-exposed mice at a FDR <30%. Clustering on the genes clearly identified two main clusters, with transcripts belonging to Cluster B being upregulated by cigarette smoking.

Functional enrichment analysis of the up- and downregulated genes, respectively, highlighted several statistically significantly (FDR <10%) enriched ontological processes of biological relevance (Table 2).

| **GO terms** | **Genes (n)** | **FDR** |
| --- | --- | --- |
| *Downregulated genes (Cluster A)* |  |  |
| GO:0006935~chemotaxis | 6 | 4.49 |
| GO:0016331~morphogenesis of embryonic epithelium | 5 | 7.88 |
| GO:0008283~cell proliferation | 8 | 9.98 |
|  |  |  |
| *Upregulated genes (Cluster B)* |  |  |
| GO:0044257~cellular protein catabolic process | 12 | 0.24 |
| GO:0007167~enzyme linked receptor protein signalling pathway | 7 | 5.61 |
| GO:0000165~MAPKKK cascade | 4 | 31.07 |
|  |  |  |

Table 2. Biological processes enriched in genes modulated by the ANOVA Group factor (FDR <30%) within each of the two main gene clusters.

We then decided to analyse each time point (that is, week 2, 12 and 24 after first exposure to CS) separately using SAM analysis [72] (Table 3). This analysis only identified 24 genes as being differentially expressed at a reasonable FDR cutoff (FDR<15%) following daily CS exposure for 24 weeks. Only by increasing the statistical cutoff to 30%, which yielded 1,020 regulated transcripts (Table 3), could we detect functional enrichment using the web-based tool DAVID (Table 4). Notable, both 2 and 12 weeks of exposure, respectively, did not cause any transcriptional changes in the mouse limb muscle (FDR <15%). For the cross-species functional comparison (see Figure 4 in manuscript) we relied on the SAM analysis (Table 3) using a FDR cutoff of 30%.

| *Duration* | **5% FDR** | **15% FDR** | **30% FDR** |
| --- | --- | --- | --- |
| 2 weeks | 0 | 0 | 5 |
| 12 weeks | 0 | 0 | 4 |
| 24 weeks | 0 | 24 (21 ↑) | 1,020 (300 ↑) |

Table S3. Number of differentially expressed genes identified by a two-class unpaired SAM analysis. The number inside the parenthesis indicates how many genes were upregulated by cigarette smoking.

| **KEGG pathways** | **Genes (n)** | **EASE**  ***P* value** | **FDR** |
| --- | --- | --- | --- |
| *Downregulated genes* |  |  |  |
| Jak-STAT signalling | 10 | 0.05 | 42% |
| TGF-beta signalling | 7 | 0.05 | 47% |
| Insulin signalling | 8 | 0.14 | 82% |
| VEGF signalling | 6 | 0.09 | 65% |
| Phosphatidylinositol signalling | 7 | 0.03 | 28% |
| Acute myeloid leukemia | 5 | 0.10 | 71% |
| Endometrial cancer | 5 | 0.08 | 61% |
| Neurotrophin signalling | 8 | 0.11 | 74% |
| Glycerophospholipid metabolism | 5 | 0.15 | 86% |
|  |  |  |  |

Table 4. Selected KEGG pathways that are enriched among the differentially expressed genes at week 24 of CS exposure (FDR <30%).

**Conclusions**

We conclude that the gastrocnemius muscle of C57 mice exposed to long-term CS (up to 24 weeks) does not show any detectable transcriptional changes at a reasonable statistical cutoff (FDR <15%).
